# Operationalizing Street Harassment Using Survey Instruments: A Systematic Review of Measuring Harassment in Public Spaces Using Surveys

**DOI:** 10.1177/15248380231219258

**Published:** 2024-02-05

**Authors:** Chloe Keel, Rebecca Stewart, Jacques Mellberg

**Affiliations:** 1 Monash University, Clayton, VIC, Australia; 2 Queensland University of Technology, Brisbane, Australia

**Keywords:** street harassment, harassment in public places, quantitative research, operationalize, survey

## Abstract

Quantitative research has an omitted variable problem when it comes to measuring and modeling non-criminal threats in the urban environment. This systematic review identified questionnaires and surveys used to measure incidences of street harassment/harassment in public, to discuss how they operationalize street harassment/harassment in public, and to report the characteristics of those with the best evidence of reliability and validity. We searched five databases and included peer-reviewed articles published in English from 1994 to 2022 that measured street harassment using a survey instrument. Our search resulted in 54 included studies. Of these studies, 16 primarily focused on understanding street harassment. To design effective prevention strategies in response to street harassment, research must first effectively measure the prevalence of street harassment and the contexts in which street harassment occurs. Due to the inconsistencies in definition, our review identified prevalence rates were inconsistent. Incidents of street harassment provide a promising avenue for future research, although scholars must first seek to appropriately operationalize this concept in survey research. We provide suggestions for future research that seeks to use surveys to understand harassment in public places.

## Introduction

Harassment in public spaces, commonly referred to as street harassment, is pervasive and a part of the everyday urban experience for certain social groups, such as women, LGBTQI+, and gender-diverse people (Ison et al. 2023; Pain, 1991, 1997; Vera-Gray, 2016). Incidents of harassment in public spaces can cause people to feel heightened fear (Stanko 1990a, 1990b, 1997; Valentine, 1992), withdrawal from their communities (Allen, 2015; Perry, 2014; Zempi & Chakraborti, 2014), and cause a decline in one’s mental and physical health (Jackson & Stafford, 2009; Zempi & Chakraborti, 2014). There is an extensive body of work that examines women’s experiences of street harassment in public places (Davis, 1994; Fileborn, 2017; Gardner, 1995; Logan, 2015; Vera-Gray, 2016). The everyday harassment of women in public spaces forms a part of the continuum of sexual and gender-based violence (Kelly, 1988). There is a theoretical proposition that incidents of harassment in public spaces are an everyday assertion of power that shapes the broader urban exchange, which may be separate from harassment in other contexts such as workplaces (Stanko 1990a, 1990b, 1997; Valentine, 1992; Vera-Gray, 2016).

Scholarly interest in street harassment has increased over the past three decades (Fileborn & O’Neill, 2021; Logan, 2015). Fileborn and O’Neill (2021) provide the most recent review of street harassment research. This review highlighted the lack of conceptual framing and definitions of street harassment used in research to date (Fileborn & O’Neill, 2021). Street harassment can include stalking, unwanted verbal attention, and unwanted physical contact, which can be sexual or non-sexual in nature (Davis, 1994; Gardner, 1995; Logan, 2015; Vera-Gray, 2016). The lack of a specific definition of street harassment has been part of the challenge for quantitative research in measuring these behaviors (see for a full review of street harassment literature Fileborn & O’Neill, 2021). In their review, Fileborn and O’Neill (2021) recommend “that researchers undertaking quantitative work develop clear definitions of what is meant by street harassment in the context of their study” (p. 10). Quantitative research has the potential to build an evidence base relating to the frequency and prevalence of street harassment; in addition, place-based context in which this harassment occurs. Emerging quantitative research has argued there is a need for developing measurement and data collection of such incidents to build an evidence regarding women’s expereinces in public spaces (Keel et al., 2023).

To date, there have been no known systematic reviews of the quantitative measurement of street harassment. Systematic reviews of measurement are important and valuable as they allow us to understand whether the field is achieving measurement validity based on current theoretical and conceptual knowledge of street harassment. Previous systematic reviews have sought to synthesize the measurement of sexual harassment across a range of settings (Ranganathan et al., 2021). This research highlighted the need for clarity in the definitions of sexual harassment to determine reliable prevalence rates. There is slippage in current research on the distinction between street harassment and sexual harassment. That is, street harassment is at times measured as sexual harassment in public places. Consistent with qualitative research which has found victims distinguish between experiences of street and sexual harassment, we argue street harassment is both a distinct form of sexual harassment and distinct from sexual harassment in that it is not always sexual in nature or motivation (Rodas-Zuleta et al., 2022). The spatial and temporal context of harassment in public places means it should not be confounded with other settings, such as workplaces and educational institutions, as often the perpetrator of street harassment is unknown to the victim and there is no clear authority to report such incidents to. Additionally, street harassment can be intersectional in nature and motivated by gender, race, class, sexual orientation, and ability (Fileborn & O’Neill, 2021). Harassment in public spaces reflects social norms and inequalities in communities that tolerate or normalize such behaviors (Cullen-Rosenthal & Fileborn, 2022). Although there is an extensive body of work on street harassment (Fileborn & O’Neill, 2021; Logan, 2015), very little is known about the prevalence or patterns of such behaviors that can be generalized to whole populations.

It has been theorized that street harassment is central to shaping individuals’ experiences and use of public spaces, particularly as it relates to perceptions of safety (Stanko 1990a, 1990b, 1997; Valentine, 1992; Vera-Gray, 2016). Consistent measurement of street harassment is needed to make comparisons over time and across different jurisdictions. At this point, it is not known whether a consistent form of measurement exists within the empirical research on street harassment. Developing consistent forms of measurement is critical to build a reliable evidence base to work toward a better understanding of street harassment as we work toward mitigating the harms of harassment and prevention of such behaviors. This paper contributes to addressing this gap by presenting a systematic review of survey instruments that measure harassment in public spaces. The objectives of this systematic review are to identify questionnaires and surveys used to measure incidences of street harassment/harassment in public, to discuss how they operationalize street harassment/harassment in public, and to report the characteristics of those with the best evidence of reliability and validity.

## Methods

### Data Sources and Search Strategy

This review was undertaken in accordance with the Preferred Reporting Items for Systematic Reviews and Meta-Analyses (PRISMA) guidelines (Moher et al., 2009; see Supplemental Appendix A for the full PRISMA Checklist). A protocol was registered on the PROSPERO International Prospective Register of Systematic Reviews in July 2022 (CRD42022344909). Given the expansive nature of the review topic, including extensive literature on sexual harassment across a wide variety of disciplines, careful consideration was given to the search terms used and the selection of databases. The search strategy and database selection were informed by the author’s experience in the fields of gender, safety, place, and quantitative methodologies, and previous experience in conducting reviews on topics related to violence prevention. The final strategy was confirmed by the second author and the lead author (see Supplemental Appendix B for a detailed search strategy). Search terms included variations on the term “street” and “public” harassment, public settings, and measurement types, in any abstract or title published in English. Title, Abstract, and Keyword filters were applied where possible, no date limitations were applied, and truncation was used in line with individual database specifications. Quantitative and mixed-method studies were identified through five electronic databases searched on July 21, 2022 (ProQuest Central, PsycINFO, Web of Science, Informit, and Google Scholar). These databases were specifically selected due to their comprehensive coverage of the area of interest across multiple relevant disciplines.

### Screening

**Figure 1.**
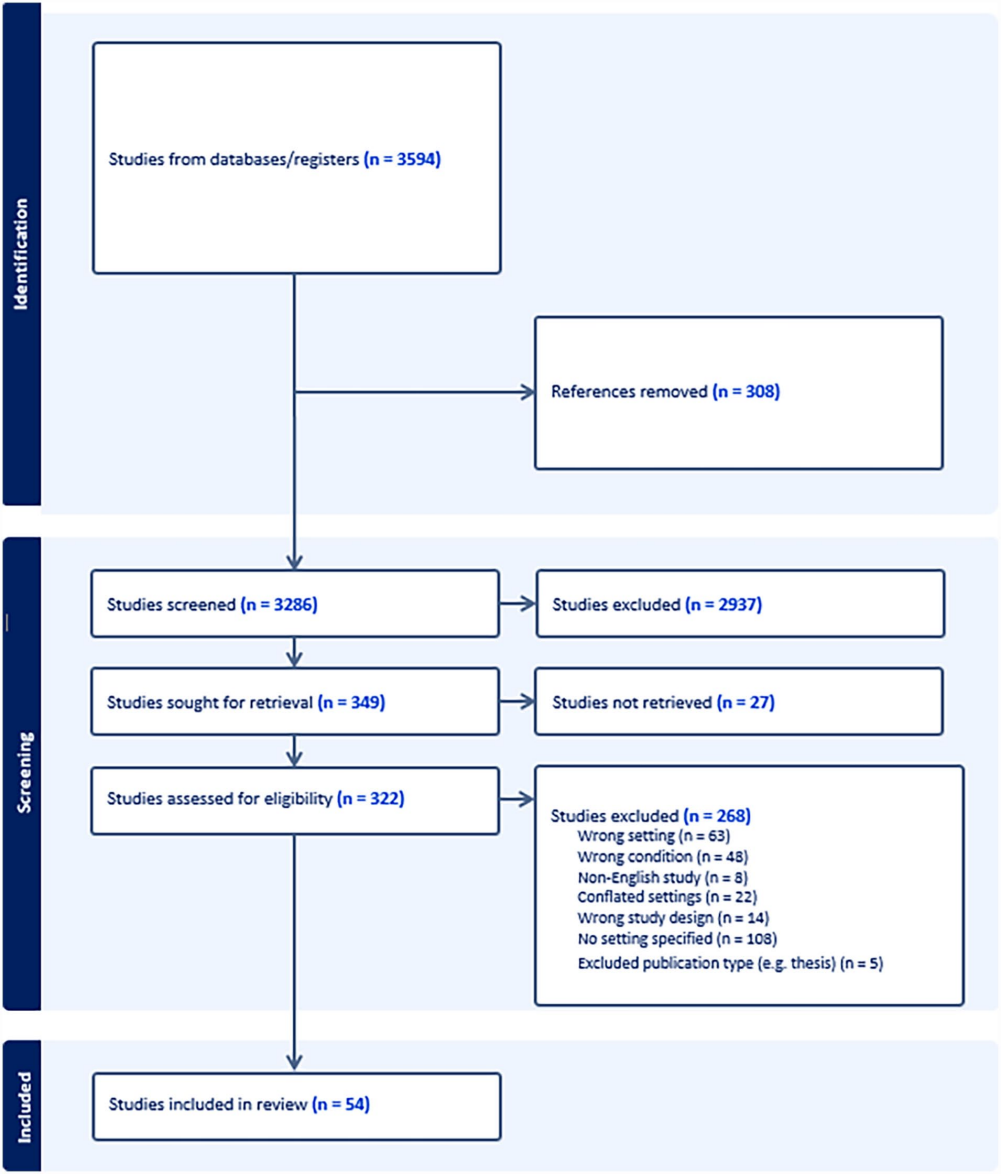
Preferred Reporting Items for Systematic Reviews and Meta-Analyses operationalizing street harassment.

Initial search results were merged and duplicates removed using EndNote before transferring data management to Covidence for screening, data extraction, and quality assessment. Two researchers independently screened titles and abstracts excluding studies based on the criteria stipulated in our PROPSPERO protocol. The University Library document request service was used to obtain articles otherwise inaccessible. For articles that did not include the measurement tool used, corresponding authors were contacted and requests were made for access to the tool. In cases where full text, measurement tools, or English versions were unable to be obtained, including non-responses from corresponding authors, the study was excluded. Full-text screening was undertaken by the same two researchers independently and the final selection resulted in 54 included studies (see Figure 1).

### Data Extraction

The data extraction template in Covidence was developed by the second author. Data extraction was undertaken by the third author and checked for accuracy by the lead author. Discrepancies were resolved by consensus between all three authors. The extracted data included the following: citation, year and location of study, participant demographics (gender, age), study design, sampling approach, delivery mode, definition of “street” or “public” harassment, measurement approach, measurement tool details including validation statistics, reporting period, disaggregation of results (by sex), prevalence rates, frequency rates, perpetrator details, and any other outcomes measured. A formal meta-analysis was not conducted given the heterogeneity of outcome variables and measures, due in part to the broad nature of the review question.

### Quality Appraisal

Using criteria adapted from Hoy et al. (2012), Ranganathan et al. (2021), and this study’s criteria, one reviewer appraised the quality of included studies. Two additional authors each then undertook an audit of 10% (*n* = 6) of studies. The completed quality appraisal table (see Supplemental Appendix C) includes 10 questions assessing study quality. This included whether studies met the review objective, the sampling strategy, description and reliability and/or validity of the measurement tool used, and prevalence rates. Each question was given a score of 0 if the answer was “no” or “unsure,” or 1 if the answer was “yes,” resulting in a maximum score of 10 points. Studies were then grouped into low quality (score of 0–3, *n* = 6), moderate quality (score of 4–6, *n* = 41), or high quality (score of 7–10, *n* = 7). Quality scores were not used to include or exclude any studies in this review.

### Data Analysis

A narrative synthesis approach was used to synthesize the data and address the aim of this study, to determine how public/street harassment is operationalized and measured. This included highlighting the variance in the quantitative literature in three key areas: definitions of street and public harassment, the types and structure of measures used, including delivery mode, and the reporting of prevalence rates. After presenting the results, we discuss the methodological quality of the included studies, in particular, the extent to which included studies spoke to the street harassment literature.

## Results

### Description of Included Studies

**Figure 2.**
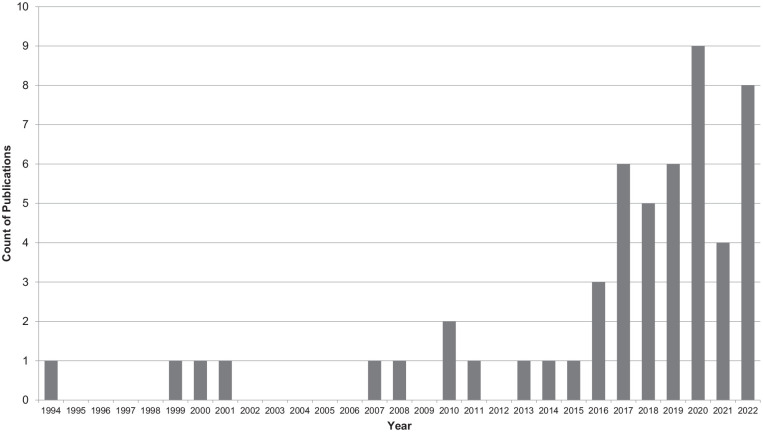
Number of included studies: measuring street harassment published between 1994 and 2022.

The characteristics of the included studies are summarized in Table 1 (see Supplemental Appendix D for full description). The earliest conducted study included in the analysis was published in 1994. Most of the studies were published between 2012 and 2022 (*n* = 45) with smaller proportions of the sample being published between 2002 and 2011 (*n* = 5) and 2001 and 1994 (*n* = 4) (see Figure 2).

**Table 1.** Summary Characteristics of Included Studies.

References	Sample		Sample Size			Definition Included		Measurement Approach			Scales		Aggregated by Sex		Perpetrator Information		Frequency of acts/experiences	
	General Population	Special Population	Less than 500	Between 500 and 5,000	Greater than 5,000	Yes	No	Direct query	Categories of Behaviors	List of behaviors or acts	Yes	No	Yes	No	Yes	No	Yes	No
Agrawal et al. (2020)		1		1		1				1		1	1			1		1
Aguilar et al. (2018)		1					1			1		1	1		1			1
Alam et al. (2010)		1			1	1		1		1		1		1	1			1
Alvi et al. (2001)		1	1				1			1	1			1		1		1
Anwar et al. (2019)		1		1			1			1	1			1	1		1	
Anwar, Osterman, Bjorkqvist et al. (2020)		1		1			1			1	1			1	1		1	
Anwar, Österman, Afari-Korkor et al. (2020)		1	1				1			1	1			1	1		1	
Awan (2020)		1	1				1			1		1		1		1	1	
Balsam et al. (2013)		1		1			1			1	1			1		1		1
Betts et al. (2018)		1	1			1		1		1		1	1			1		1
Brewster et al. (2019)		1	1				1	1		1	1			1		1	1	
Campos et al. (2017)		1		1			1	1		1		1		1	1			1
Carretta and Szymanski (2020)		1	1			1				1	1			1		1	1	
Ceccato and Loukaitou-Sideris (2021)		1			1	1				1		1	1			1		1
Ceccato and Loukaitou-Sideris (2022)		1			1	1				1		1	1			1		1
Davidson et al. (2015)		1		1		1				1	1			1		1	1	
Davidson et al. (2016)		1		1		1				1	1			1		1	1	
del Mar Rodas-Zuleta et al. (2022)	1		1			1		1		1		1	1			1		1
DelGreco and Christensen, (2020)		1	1			1				1	1			1		1	1	
DelGreco et al. (2021)		1	1			1				1		1		1	1		1	
Doan (2007)		1	1				1	1		1		1	1			1		1
Emerson et al. (2016)	1				1		1			1		1		1		1		1
Fairchild (2008)		1	1			1				1	1			1		1	1	
Fairchild (2010)		1	1	1		1				1		1		1		1	1	
Ferrer-Perez (2021)		1		1		1		1				1	1			1	1	
Fileborn (2019)		1	1			1		1				1		1	1		1	
Gurrola-Peña et al. (2022)		1	2^a^			1				1	1			1		1	1	
Heesch (2011)		1		1			1	1		1		1	1			1		1
Imtiaz (2021)		1	1			1				1	1		1		1		1	
Infante-Vargas (2022)		1		1		1				1		1		1		1	1	
Jabeen et al. (2017)		1		1		1		1		1		1		1	1		1	
Kash (2019)		1	1^c^			1				1		1		1		1	1	
Kearl (2014)	1			1^b^		1				1		1	1		1		1	
Khairat (2016)		1	1			1		1	1			1	1		1		1	
Lebugle (2017)	1				1	1				1		1	1		1		1	
Lenton et al. (1999)		1		1			1			1		1	1			1	1	
Loukaitou-Sideris (2022)		1			1	1				1		1	1			1		1
Loukaitou-Sideris et al. (2020)		1		1		1				1		1	1			1		1
Macmillan et al. (2000)		1			1	1				1		1		1		1		1
Malik et al. (2020)		1	1				1	1	1			1		1		1		1
Marmet (2017)	1			1^b^			1	1		1		1		1	1		1	
Mellgren et al. (2018)		1		1		1		1				1		1	1		1	
Mishra (2018)		1	1			1		1	1			1		1	1		1	
Mora et al. (2022)		1	1			1				1	1			1		1	1	
Moreno et al. (2022)	1				1	1				1	1			1		1		1
Natarajan et al. (2017)		1	1				1	1				1		1		1	1	
Raj et al. (2021)	1			1			1			1		1	1			1		1
Reed et al. (2019)		1	1			1				1	1			1	1		1	
Saunders et al. (2017)		1	1			1				1	1			1		1	1	
Shibata (2020)		1	1				1			1		1	1			1		1
Smith (1994)	1			1		1		1				1		1	1		1	
Smith et al. (2022)	1			1		1				1		1	1		1			1
Solymosi et al. (2018)	1		1			1				1		1		1		1		1
Whitfield et al. (2019)		1		1			1	1				1	1		1		1	
Total	10	44	26	21	8	35	19	18	3	45	17	37	20	34	18	36	30	24

a Denotes multiple samples.

b Denotes unweighted sample.

c Denotes subsample which were asked street harassment items, original *n* = 1,444.

The most common location where data about street harassment were collected was the United States of America (*n* = 19), followed by Pakistan (*n* = 5), Mexico (*n* = 4), and Canada (*n* = 4). It is worth noting that *n* = 6 studies included in the analysis report findings from a larger survey conducted in multiple countries (Agrawal et al., 2020; Ceccato & Loukaitou-Sideris, 2021, 2022; Loukaitou-Sideris & Ceccato, 2022; Loukaitou-Sideris et al., 2020; Shibata, 2020). One study collected survey data from an internet sample, with no geographic specification (Fairchild, 2010).

All of the articles included in the analysis used cross-sectional datasets (*n* = 54). Those that drew upon longitudinal datasets only analyzed one wave (Emerson et al., 2016; Mora et al., 2022), resulting in a cross-sectional analysis. One study used a quasi-experimental design; however, the outcome variable was not experiences of street harassment (Fairchild, 2010).

There were comparatively few studies that collected data from general population samples (*n* = 10), with most studies drawing upon subpopulations (*n* = 44). The most common subpopulation was university or college students (*n* = 16). Further information about subpopulations is provided later in the results.

Sample sizes for the studies included in the analysis ranged from 118 to 41,895 respondents (Mdn = 540.5, *SD* = 7,241.30). Almost all of the studies included in the analysis reported findings from a single dataset (*n* = 52). Of these, most had sample sizes smaller than 500 respondents (*n* = 23), a similar number of studies had medium sample sizes of between 500 and 5,000 respondents (*n* = 20). A small proportion of studies contained more than 5,000 respondents (*n* = 9).

There were two studies that drew upon multiple datasets. The first had two samples of smaller than 500 respondents. The second study had a sample of between 500 and 5,000 respondents for the first study and a sample smaller than 500 respondents for the second study. Few studies had a sample that represented the population they sought to study (*n* = 10). Two studies did not provide sufficient information to make a judgment about representativeness.

### Definition of Street Harassment

Although most studies (*n* = 35) included some form of definition of street harassment, the nature of these definitions varied quite substantially. Only *n* = 16 studies explicitly mentioned “stranger harassment” or “street harassment” in their definition. The remaining studies (*n* = 19) provided definitions for sexual harassment, sexual assault, and/or gender-based harassment. Regardless of whether or not “street harassment” or “stranger harassment” were mentioned, street harassment was most commonly defined as sexual or gender-based harassment that occurs in public places and is perpetrated by strangers. The definitions often included lists of behaviors as examples, such as whistling, catcalling, leering, unwanted conversation, and unwanted physical touch. In some cases, only lists of behaviors were provided as a definition. The lists of behaviors were often categorized at the conceptualization phase of the study into verbal, physical, and non-verbal/non-physical, in line with Fitzgerald et al.’s (1995) Sexual Harassment Experiences Questionnaire. A full summary of the definitions has been provided in Supplemental Appendix E.

Very few studies explicitly distinguished street harassment from other forms of harassment (*n* = 3). Explanations for street harassment being different from other forms of harassment included the public location in which the harassment occurred (Kearl, 2014; Saunders et al., 2017), that the harassment may not always contain a sexual component (Rodas-Zuleta et al., 2022), the absence of a perpetrator’s prior relationship to the victim (Saunders et al., 2017), and differences in motivation for the perpetrator (Saunders et al., 2017).

### Measurement Approach for Street Harassment

The most common measurement approach for capturing street harassment was providing participants with a list of behaviors (e.g., which of the following behaviors have you ever experienced?; *n* = 49), followed by a direct query (e.g., have you ever been sexually harassed in public?; *n* = 16) and categories of behaviors (e.g., verbal harassment; *n* = 3). Among the studies that employed the list of behaviors approach (*n* = 49), 30 studies provided participants with binary response options (e.g., yes or no) to indicate whether or not they had experienced each act. Nineteen of the studies that employed the list of behaviors approach to measure street harassment provided participants with response alternatives that allowed them to indicate the frequency of their experiences (e.g., 0 = Never, 1 = Once in the past year, 2 = A few times in the past year, 3 = About once a month, 4 = A few times a month, 5 = Almost every day, and 6 = Multiple times a day [DelGreco & Christensen, 2020]).

The majority of studies (*n* = 41) used a single approach to measure street harassment. Of these, most (*n* = 38) used lists of behaviors, while few studies relied solely on a direct query approach (*n* = 3). A small number of studies (*n* = 13) used multiple approaches to measure street harassment. Among these studies, two measured street harassment using categories of behaviors and a direct query approach, one study used all three approaches, while the remaining multiple-approach studies (*n* = 10) used direct queries and lists of behaviors/acts.

Overall, there was substantial heterogeneity within the approaches to measuring street harassment. The most common reporting period for the survey items measuring street harassment was over the course of the participant’s lifetime (i.e., ever; *n* = 27), followed by within the previous 12 months (*n* = 11), within the previous 3 years (*n* = 6), within the previous 6 months (*n* = 2), within the previous month (*n* = 1), while on holiday (*n* = 1), and while using transport (*n* = 1). One study asked participants to report their experiences both within their lifetime and within the previous 12 months (Infante-Vargas, 2022). One study asked participants to report their experiences immediately after each incident had occurred (Betts et al., 2018). There were *n* = 3 studies that did not provide sufficient information to make a judgment. A full description of the measurement tool used can be found in Supplemental Appendix D.

The current review also assessed whether each study reported the results of a quality appraisal of their survey instrument, including assessments of face validity, test–retest reliability, convergent validity, and/or congeneric reliability (e.g., Cronbach’s alpha). The majority of studies included in this review did not report any form of quality assessment of their survey instrument (*n* = 37). The remaining studies that included a quality check of their survey instruments all reported Cronbach’s alpha (*n* = 17). A small handful of these studies also reported the results of factor analyses (*n* = 4). The latent variables identified from the factor analyses did not specifically focus on “street harassment” but rather related concepts including discrimination/harassment (Balsam et al., 2013), physical harassment, verbal harassment (Fairchild, 2008), and sexual harassment and non-sexual harassment (Gurrola-Peña et al., 2022).

### Prevalence of Street Harassment

While 76% of studies included a prevalence rate, there was a lack of consistent definition and measurement of street harassment across the included studies. This made it challenging to determine prevalence rates. Table 2 provides prevalence measures, scale, and special population. In studies that used only a direct query method, the prevalence of street harassment (as defined by the study) ranged from 20.22% to 95.4%. Among studies where they used behaviors or acts, the prevalence ranges from 17% to 91%. There was a higher prevalence rate for non-physical forms of harassment in public spaces. For example, the range for verbal comments was 17% to 85% compared to the range for unwanted touching which was 28% to 54%. In studies with special populations (*n* = 44), male university students reported the lowest prevalence of street harassment 2.6% to 65%. Adult females (12%–80.9%) and female university students (11.2%–87.6%) experienced similar rates, while LGBTQI+ respondents reported the highest level (17%–95%).

**Table 2.** Prevalence of Street Harassment by Measures and Population.

Type	Prevalence Range	Study Reference/s
Measurement approach		
Direct query method	20.2%–95.4%	Alam et al. (2010), Betts (2019), Brewster et al. (2019), Campos et al. (2017), del Mar Rodas-Zuleta et al. (2022), Doan (2007), Ferrer-Perez (2021), Fileborn (2019), Gurrola-Peña et al. (2022), Infante-Vargas (2022), Kearl (2014), Macmillan et al. (2000), Malik et al. (2020), Marmet (2017), Mellgren et al. (2018), Moreno et al. (2022), Shibata (2020), Solymosi et al. (2018)
List of behaviors or acts (wide-ranging behaviors). Most common below:		Agrawal et al. (2020), Aguilar et al. (2018), Alam et al. (2010), Alvi et al. (2001), Anwar, Österman, Björkqvist et al. (2020), Awan (2020), Balsam et al. (2013), Betts (2019), Brewster et al. (2019), Campos et al. (2017), Carretta and Szymanski (2020), Ceccato and Loukaitou-Sideris (2021, 2022), Davidson et al. (2015, 2016), del Mar Rodas-Zuleta et al. (2022), DelGreco and Christensen, (2020), DelGreco et al. (2021), Doan (2007), Emerson et al. (2016), Fairchild (2008, 2010), Gurrola-Peña et al. (2022), Heesch (2011), Imtiaz (2021), Infante-Vargas (2022), Jabeen et al. (2017), Kash (2019), Khairat (2016), Lebugle (2017), Lenton et al. (1999), Loukaitou-Sideris et al. (2020), Loukaitou-Sideris (2022), Malik et al. (2020), Mishra (2018), Mora et al. (2022), Natarajan et al. (2017), Raj et al. (2021), Reed et al. (2019), Saunders et al. (2017), Smith (1994), Smith et al. (2022), Whitfield et al. (2019)
Verbal comments	17%–85%	Campos et al. (2017), Doan (2007)
Staring/stalking/following	22%–91%	Campos et al. (2017)
Unwanted touching	28%–54%	Campos et al. (2017)
Special populations		
Females	12%–80.9%	Campos et al. (2017), Fairchild (2010), Jabeen et al. (2017), Macmillan et al. (2000), Moreno et al. (2022), Lenton et al. (1999), Solymosi et al. (2018)
LGBTQI+	17%–95.1%	Doan (2007), Whitfield et al. (2019)
Female university students	11.2%–87.6%	Davidson et al. (2016), Fairchild (2008), Mishra (2018), Mellgren et al. (2018), Natarajan et al. (2017), Saunders et al. (2017)
Adolescents	27%–65%	Betts (2019), Ferrer-Perez (2021), Reed et al. (2019), Mora et al. (2022)
University students (both sexes)	M: 2.6%–65%—W: 24.1%–92% A; 26%–92%	Agrawal et al. (2020), Ceccato and Loukaitou-Sideris (2021, 2022), Loukaitou-Sideris et al. (2020), Loukaitou-Sideris and Ceccato (2022), Shibata (2020)

## Discussion

Quantitative research that seeks to understand experiences in and perceptions of public places has an omitted variable problem (Keel et al., 2023). That is, it is leaving out an important factor in the analysis of experiences and perceptions of place, street harassment. While previous systematic reviews have focused on sexual harassment across all contexts (Ranganathan et al., 2021), street harassment warrants a specific review due to the public setting in which the harassment occurs and the intersectional motivations of the harassment, which can be sexual or non-sexual. Qualitative studies have highlighted street harassment as an emerging area of research over the past 30 years, yet, there is limited quantitative research in the field (Fileborn & O’Neill, 2021; Logan, 2015). It is clear from our review that a quantitative focus is more recent, with the majority of quantitative studies conducted in the past 6 years. Below we discuss the key findings from the systematic review as it relates to the current conceptualization and operationalization of street harassment in survey research. A summary of critical findings and implications for practice, policy, and research is found in Table 3.

**Table 3.** Critical Findings and Implications for Practice, Policy and Research.

Issue	Critical Findings	Implications for Practice, Policy, Research
Conceptualization of street harassment	• There is no universally agreed upon definition of street harassment within the quantitative scholarship. • A number of definitions refer to sexual assault, or other criminal acts, in public spaces.	• Researchers should establish a clear conceptual definition of street harassment. • The definition should account for the intersectional nature of street harassment as a behavior women experience but also other social groups.
Operationalization of street harassment	• There is substantial heterogeneity in the measurement of street harassment using quantitative tools. • The most common approach is to provide participants with a list of behaviors or acts which are considered to be a type of street harassment. • Few studies draw upon nationally representative samples. • Overall, it is difficult to gain an understanding of what street harassment looks like at an incident level from the quantitative scholarship.	• The prevalence and perceptions of street harassment should be operationalized separately to guide policy and practice. • Street harassment surveys should draw upon nationally representative samples. • Surveys measuring the prevalence of street harassment should seek to capture details about “who, what, when, and where,” including perpetrator details. • Studies would benefit from employing Ecological Momentary Assessments to capture street harassment and triangulate quantitative data with qualitative findings.

While almost all studies in this review provided a definition, of harassment, only 16 studies explicitly spoke to street harassment. Notably, 19 studies within our sample presented conceptual definitions of sexual harassment, sexual assault, and/or gender-based harassment as their definition of harassment. This overlapping conceptual definition may be reflected by the majority of studies that employed Fitzgerald et al.’s (1995) measure of sexual harassment, the Sexual Experiences Questionnaire, and asked about the different contexts in which the harassment had occurred. The lack of a clear conceptualization of street harassment is reflective of the findings of Fileborn and O’Neill (2021), speaking to the lack of a clear definition of street harassment as a phenomenon in its own right, particularly within the quantitative literature.

Existing qualitative research, from which the quantitative scholarship on street harassment has emerged, argues that street harassment is a part of the continuum of gendered violence (Kelly, 1988), it is also intersectional, and includes the harassment of other groups, and is not always sexual (Fileborn & O’Neill, 2021). Our review finds that the quantitative tools used to measure street harassment currently do not clearly reflect these lived experiences. There were a number of definitions offered in the scholarship that defined the victim (a woman), the place (the street), the perpetrator’s relationship to the victim (a stranger or unknown man), and the behavior (unwanted or unwelcome). For example, LGBTQI+ and gender-diverse people’s experiences may be missed in studies that exclusively focus on women (Kolysh, 2021) and migrant and refugee women’s experiences may be limited without a definition that allows for gendered and racial motivations to the harassment (Mason-Bish & Zempi, 2018). Harassment in public places is distinct, in part due to the unique spatial and temporal context in which it occurs, as well as the intersecting motivations and manifestations. Moving forward studies that seek to conceptualize and operationalize incidents of harassment must consider the unique nature of harassment in public places. We recommend quantitative research establish a clear conceptual definition of street harassment informed by the qualitative learnings in the literature.

Quantitative surveys are effective tools for capturing patterns and characteristics of victimization experiences (Lynch & Addington, 2010). To date literature has been unable to provide a generalizable account of street harassment incidents, such as the location in which they occur (i.e., how far from home the incident occurred, types of places street harassment might occur) and who the harasser is (i.e., the gender of the harasser, are they know to the individual). The studies and survey instruments captured in this review indicate a current preference in the literature for the measurement of the frequency of experiences as opposed to a description of harassment in public places. This focus makes it difficult to ascertain what an incident of street harassment might look like due to a paucity of data collected in relation to the location where the harassment took place, as well as a lack of information captured about perpetrators of street harassment. To measure street harassment, it is important to investigate the “descriptive” information associated with the incidents. Specifically, it is recommended that details about the perpetrator/s and the presence of bystanders are captured (who). Researchers should also seek to identify ways to collect data about street harassment acts that occur at the same time (what). For example, an incident may include being verbally harassed and physically touched in the same incident. Given the importance of temporal and spatial contexts for street harassment, survey instruments should also aim to collect information about the time of day and location in which the street harassment incident occurred (when and where). A small proportion of studies within our review sought to capture this information from participants. Notably, Fileborn’s (2019) survey instrument asked participants whether they had been harassed in Melbourne, Victoria, Australia. Those participants who selected “yes” were prompted to describe their experiences in terms of what happened, when it happened, and where it happened. In addition to these single-incident measures, participants were asked to provide the estimated frequency of their experiences. The combination of single-incident measures and frequency estimates may provide researchers with a clearer picture of what an incident of street harassment looks like and how often these incidents occur. By asking participants to describe their experiences of street harassment, more informed policy decisions can be made about how to best respond to and prevent these incidents.

The current review initially sought to assess whether the quantitative scholarship on street harassment regularly reported quality assessment of their survey instruments. However, as noted in the results section of this review, there was substantial heterogeneity in the approaches to measuring street harassment. The current review is, therefore, unable to definitively assess the overall reliability and validity of the quantitative survey instruments used to measure street harassment. To work toward a better understanding of “what works” in the field, researchers first need to arrive at a consensus on how to best conceptualize street harassment. In doing so, more consistent measurement approaches may be developed which can be tested across geographic and social contexts. Furthermore, an assessment of street harassment’s overall prevalence was unable to be estimated in this review. To ensure the generalizability of prevalence estimates, researchers also need to draw upon non-probability random samples that are representative. Within our review, only 15 studies employed probability sampling approaches that could be generalized to the broader population.

As the quantitative scholarship on street harassment continues to develop, stable correlates of experiences of street harassment may be identified. Researchers may then assess the predictive validity of their street harassment survey measures. Predictive validity allows researchers to assess whether a construct measured by a survey instrument is a stable predictor or a correlate of another variable across studies (Hughes, 2018). However, this approach requires consistent approaches to measurement and representative samples (Hughes, 2018), which were generally rare within the studies included in the current review. In light of the varied approaches to conceptualizing street harassment, researchers developing new survey instruments should consider greater use of pilot testing, assessments of face validity, and triangulation of participants’ experiences with qualitative data. These approaches may provide a clearer understanding of how participants interpret the behaviors and acts included in survey instruments measuring street harassment.

In addition, people might categorize their experiences of street harassment using different factors such as how distressing the experience was, what the perceived motivation for the behavior was, or whether there were multiple contemporaneous acts of harassment involved. Within our review, very few studies explored these perceptual measures associated with street harassment. Notably, Balsam et al.’s (2013) survey asked participants to indicate whether they had experienced an act of harassment and to what extent the experience bothered them. In doing so, Balsam et al. (2013) were able to estimate the prevalence and distress of participants’ experiences using a single set of survey items. Future research might extend upon Balsam et al.’s (2013) approach to develop a survey that primarily focuses on measuring street harassment. Scholars developing this tool may also consider the perceived motivation of the incident to better understand the intersectional nature of street harassment. Overall, scholars should seek to distinguish, theoretically and methodologically, the prevalence of street harassment and victims’ perceptions of street harassment. In doing so, richer information about the nature of people’s experiences with street harassment can be gleaned with more targeted approaches for managing its prevalence and consequences for individuals and communities.

Emerging quantitative data collection techniques in the social sciences such as Ecological Momentary Assessments (EMAs) may assist with participant recall and capture a more comprehensive picture of these incidents (Shiffman et al., 2008). EMAs describe approaches to data collection that use repeated assessments to capture participants’ experiences in real time and their natural environments (Shiffman et al., 2008). EMAs may take the form of paper-pencil diary entries or online survey instruments delivered via mobile devices and potentially offer researchers a granular understanding of participants’ experiences by asking each individual to report their experiences in real time. As noted in the results section of this review, the most common reporting period for survey instruments measuring street harassment was the participants’ lifetime followed by within the past 12 months. As an alternative, EMAs could allow researchers to more easily collect incident-level data about street harassment by prompting participants to complete a survey within a short timeframe after an experience of harassment.

Finally, to facilitate the future directions outlined above, it is important that the scholarship looks to improve the public availability of their survey measures. The majority of studies did not include reproducible research and there was limited publicly available data on research design. We argue that this is may limit the opportunity for meaningful improvements to methodology and survey design to be made.

### Limitations

While this review has provided the first comprehensive review of street harassment measurement in surveys, there are three key limitations to note. First, it was evident from our review that research is emerging from non-English literature, in particular from India and Latin American countries. The exclusion of non-English literature in our review may cause potential representations of non-English-speaking countries. Furthermore, street harassment in India is colloquially referred to as “eve-teasing”; however, we did not include this term in our search strategy and as a result may have missed survey instruments emerging from India. Future reviews should examine the way in which non-English studies measure street harassment. A second limitation relates to the assessments of the methodological qualities of the studies. The aim of this review was to critique the current operationalization and measurement of street harassment in the quantitative literature. As such we did not exclude studies based on quality, nor the provision or absence of a measurement validation tool. However, we acknowledge that the use of low-, medium-, and high-quality assessments may incorrectly imply equal weight for each of the quality criteria. This could arguably misclassify some studies as it relates to their overall quality. The third limitation of our review is the heterogeneity of definitions of harassment in public places. It is evident from this review there is a lack of consistent understanding relating to what constitutes street harassment and who perpetrates it. This includes the frequent ambiguity in the included papers around the location of harassment—which included the exclusion of articles when the location was not clearly articulated, potentially resulting in measures that actually do measure this phenomenon being missed. Furthermore, there was definitionally complexity around the relationship between street harassment with other forms of sexual violence by some studies. If street harassment was a secondary objective of a study and did not feature in the title or abstract, it may not have been captured in our included studies resulting in publication bias.

## Conclusion

To design effective prevention strategies in response to street harassment, research must first effectively measure the prevalence of street harassment and the contexts in which street harassment occurs. Due to the inconsistencies in definition, our review identified prevalence rates were inconsistent. In addition to consistent definition, studies that examine street harassment should seek to capture details about the perpetrator. Our review found that 18 studies included details about the perpetrator. In light of the momentary nature of street harassment, greater information about who perpetrates street harassment and in what context will be of great use when understanding why it occurs and how to prevent it. To date, very little is known about perpetration. Current prevalence rates of street harassment are often not societally representative and tend to focus on smaller cohorts of the community. Given the broad spectrum of social groups that experience street harassment, a need for a systematic approach to data collection and measurement is clear. The growing evidence base points to the significance of harassment in public spaces and highlights the need to develop a clear conceptualization and operationalization of harassment in public spaces.

## Supplemental Material

sj-doc-1-tva-10.1177_15248380231219258 – Supplemental material for Operationalizing Street Harassment Using Survey Instruments: A Systematic Review of Measuring Harassment in Public Spaces Using Surveys Supplemental material, sj-doc-1-tva-10.1177_15248380231219258 for Operationalizing Street Harassment Using Survey Instruments: A Systematic Review of Measuring Harassment in Public Spaces Using Surveys by Chloe Keel, Rebecca Stewart and Jacques Mellberg in Trauma, Violence, & Abuse

sj-docx-2-tva-10.1177_15248380231219258 – Supplemental material for Operationalizing Street Harassment Using Survey Instruments: A Systematic Review of Measuring Harassment in Public Spaces Using Surveys Supplemental material, sj-docx-2-tva-10.1177_15248380231219258 for Operationalizing Street Harassment Using Survey Instruments: A Systematic Review of Measuring Harassment in Public Spaces Using Surveys by Chloe Keel, Rebecca Stewart and Jacques Mellberg in Trauma, Violence, & Abuse

sj-docx-3-tva-10.1177_15248380231219258 – Supplemental material for Operationalizing Street Harassment Using Survey Instruments: A Systematic Review of Measuring Harassment in Public Spaces Using Surveys Supplemental material, sj-docx-3-tva-10.1177_15248380231219258 for Operationalizing Street Harassment Using Survey Instruments: A Systematic Review of Measuring Harassment in Public Spaces Using Surveys by Chloe Keel, Rebecca Stewart and Jacques Mellberg in Trauma, Violence, & Abuse

sj-docx-4-tva-10.1177_15248380231219258 – Supplemental material for Operationalizing Street Harassment Using Survey Instruments: A Systematic Review of Measuring Harassment in Public Spaces Using Surveys Supplemental material, sj-docx-4-tva-10.1177_15248380231219258 for Operationalizing Street Harassment Using Survey Instruments: A Systematic Review of Measuring Harassment in Public Spaces Using Surveys by Chloe Keel, Rebecca Stewart and Jacques Mellberg in Trauma, Violence, & Abuse

sj-docx-5-tva-10.1177_15248380231219258 – Supplemental material for Operationalizing Street Harassment Using Survey Instruments: A Systematic Review of Measuring Harassment in Public Spaces Using Surveys Supplemental material, sj-docx-5-tva-10.1177_15248380231219258 for Operationalizing Street Harassment Using Survey Instruments: A Systematic Review of Measuring Harassment in Public Spaces Using Surveys by Chloe Keel, Rebecca Stewart and Jacques Mellberg in Trauma, Violence, & Abuse
