# Supplementary material for: Operationalizing Street Harassment Using Survey Instruments: A Systematic Review of Measuring Harassment in Public Spaces Using Surveys
Source: Trauma Violence Abuse. 2024 Feb 5;25(4):2609–21. doi: 10.1177/15248380231219258 (PMC11370194; doi:10.1177/15248380231219258)
Supplement: sj-docx-2-tva-10.1177_15248380231219258 – Supplemental material for Operationalizing Street Harassment Using Survey Instruments: A Systematic Review of Measuring Harassment in Public Spaces Using Surveys [file sj-docx-2-tva-10.1177_15248380231219258.docx]

| **Search Engine** | **Search Strategy** |
| --- | --- |
| ProQuest | - String 1: harass* OR "sexual harass*" OR "street sexual harass*" OR "sexual street harass*" OR “public incivilit*” OR “public harassment” OR “street harass*”   AND   - String 2: street OR “public space*” OR neighbo?rhood* OR "public transport" OR outdoor* OR outside   AND   - String 3: survey OR questionnaire OR measur* OR scale |
| PsycINFO | Search each line individually & apply the filters ‘all journals’, ‘English language’ and ‘Human’ for each search:   - Harassment or incivility - Street or public or sexual or public transport or neighbourhood or outdoor or outside - survey OR questionnaire OR measur* OR scale |
| Web of Science | ((ALL=(harass* OR "sexual harass*" OR "street sexual harass*" OR "sexual street harass*" OR “public incivilit*” OR “public harass*” OR "street harass*")) AND ALL=(street OR “public space*” OR neighbo?rhood* OR "public transport" OR outside OR outdoor*)) AND ALL=(survey OR questionnaire OR measur* OR scale) |
| Informit | - harassment - street OR public OR sexual - survey OR questionnaire OR measur* OR scale |
| Google Scholar | (harassment OR sexual harassment OR sexual street harassment OR street sexual harassment OR public incivilities OR public harassment OR street harassment) AND (public spaces OR street OR neighbourhood OR public transport OR outdoors OR outside) AND (survey OR questionnaire OR measure) |
