# Supplementary material for: Operationalizing Street Harassment Using Survey Instruments: A Systematic Review of Measuring Harassment in Public Spaces Using Surveys
Source: Trauma Violence Abuse. 2024 Feb 5;25(4):2609–21. doi: 10.1177/15248380231219258 (PMC11370194; doi:10.1177/15248380231219258)
Supplement: sj-docx-3-tva-10.1177_15248380231219258 – Supplemental material for Operationalizing Street Harassment Using Survey Instruments: A Systematic Review of Measuring Harassment in Public Spaces Using Surveys [file sj-docx-3-tva-10.1177_15248380231219258.docx]

Each question was given a score of 0 (low) if the answer was ‘no’ or ‘unsure’ or 1 (high) if the answer was ‘yes’, resulting in a maximum score of 10 points. Studies with were then grouped into low quality (score of 0-3), moderate quality (score of 4-6) or high quality (score of 7-10). Quality scores were not used to include or exclude any studies in this review.

Questions:

1. Research focus on street or public harassment
2. Definition of street / public harassment
3. Description of measurement tool
4. Representativeness of target population
5. Representativeness of sampling frame
6. Random sampling
7. Non-response bias
8. Harassment prevalence
9. Reliability and validity of measurement tool
10. Clear & appropriate numerator & denominator

| **Study Author, Year** | **Q1** | **Q2** | **Q3** | **Q4** | **Q5** | **Q6** | **Q7** | **Q8** | **Q9** | **Q10** | **Total /10** | **Study Quality** |
| --- | --- | --- | --- | --- | --- | --- | --- | --- | --- | --- | --- | --- |
| Agrawal et al., 2020 | 1 | 1 | 1 | 0 | 1 | 0 | 0 | 1 | 0 | 1 | 6 | Moderate |
| Aguilar et al., 2018 | 1 | 0 | 1 | 1 | 1 | 1 | 0 | 1 | 0 | 1 | 7 | High |
| Alam at al., 2010 | 1 | 0 | 1 | 1 | 1 | 0 | 0 | 1 | 0 | 1 | 6 | Moderate |
| Alvi et al., 2001 | 1 | 0 | 1 | 0 | 0 | 1 | 0 | 0 | 0 | 0 | 3 | Low |
| Anwar et al., 2019 | 1 | 0 | 1 | 0 | 0 | 1 | 0 | 0 | 1 | 1 | 5 | Moderate |
| Anwar, Österman, Björkqvist, 2020 | 1 | 0 | 1 | 0 | 0 | 1 | 0 | 0 | 1 | 1 | 5 | Moderate |
| Anwar, Österman, Afari-Korko, Björkqvist,  2020 | 1 | 0 | 1 | 0 | 0 | 1 | 0 | 0 | 1 | 1 | 5 | Moderate |
| Awan, 2020 | 1 | 0 | 0 | 0 | 0 | 0 | 0 | 1 | 0 | 1 | 3 | Low |
| Balsam et al., 2013 | 1 | 0 | 0 | 0 | 0 | 1 | 0 | 0 | 1 | 1 | 4 | Moderate |
| Betts et al., 2019 | 1 | 1 | 1 | 0 | 0 | 1 | 0 | 1 | 0 | 1 | 6 | Moderate |
| Brewster et al., 2019 | 1 | 0 | 1 | 0 | 0 | 1 | 1 | 0 | 1 | 0 | 5 | Moderate |
| Campos et al., 2017 | 1 | 0 | 0 | 0 | 0 | 0 | 0 | 1 | 0 | 1 | 3 | Low |
| Carretta et al., 2020 | 1 | 1 | 1 | 0 | 0 | 1 | 0 | 1 | 1 | 0 | 6 | Moderate |
| Ceccato et al., 2021 | 1 | 1 | 0 | 0 | 0 | 1 | 0 | 1 | 0 | 1 | 5 | Moderate |
| Ceccato et al., 2022 | 1 | 1 | 0 | 0 | 0 | 1 | 0 | 1 | 0 | 0 | 4 | Moderate |
| Davidson et al., 2015 | 1 | 1 | 1 | 0 | 0 | 1 | 0 | 0 | 1 | 1 | 6 | Moderate |
| Davidson et al., 2016 | 1 | 1 | 1 | 0 | 0 | 1 | 0 | 1 | 1 | 0 | 6 | Moderate |
| del Mar Rodas-Zuleta et al., 2022 | 1 | 1 | 0 | 0 | 1 | 1 | 0 | 1 | 0 | 0 | 5 | Moderate |
| DelGreco et al., 2020 | 1 | 1 | 0 | 0 | 0 | 1 | 0 | 0 | 1 | 0 | 4 | Moderate |
| DelGreco et al., 2021 | 1 | 1 | 0 | 0 | 0 | 1 | 0 | 0 | 0 | 0 | 3 | Low |
| Doan, 2007 | 1 | 0 | 1 | 0 | 0 | 1 | 0 | 1 | 0 | 1 | 5 | Moderate |
| Emerson et al., 2016 | 1 | 0 | 1 | 1 | 1 | 0 | 0 | 1 | 0 | 1 | 6 | Moderate |
| Fairchild et al., 2008 | 1 | 1 | 1 | 0 | 0 | 1 | 0 | 1 | 1 | 0 | 6 | Moderate |
| Fairchild, 2010 | 1 | 1 | 1 | 0 | 0 | 1 | 0 | 1 | 0 | 0 | 5 | Moderate |
| Ferrer-Perez et al., 2021 | 1 | 1 | 1 | 0 | 0 | 1 | 0 | 1 | 0 | 1 | 6 | Moderate |
| Fileborn, 2019 | 1 | 1 | 0 | 0 | 0 | 1 | 0 | 0 | 0 | 1 | 4 | Moderate |
| Gurrola-Peña et al., 2022 | 1 | 1 | 0 | 0 | 0 | 1 | 0 | 0 | 1 | 0 | 4 | Moderate |
| Heesch et al., 2011 | 1 | 0 | 1 | 0 | 0 | 1 | 0 | 1 | 0 | 1 | 5 | Moderate |
| Imtiaz et al., 2021 | 1 | 1 | 0 | 0 | 0 | 1 | 0 | 1 | 1 | 1 | 6 | Moderate |
| Infante-Vargas et al., 2022 | 1 | 1 | 0 | 0 | 0 | 1 | 0 | 1 | 0 | 1 | 5 | Moderate |
| Jabeen et al., 2017 | 1 | 1 | 0 | 0 | 0 | 1 | 0 | 1 | 0 | 1 | 5 | Moderate |
| Kash, 2019 | 1 | 1 | 1 | 0 | 1 | 0 | 0 | 1 | 0 | 0 | 5 | Moderate |
| Kearl, 2014 | 1 | 1 | 1 | 1 | 1 | 1 | 0 | 1 | 0 | 1 | 8 | High |
| Khairat, 2016 | 1 | 1 | 0 | 0 | 0 | 1 | 0 | 1 | 0 | 1 | 5 | Moderate |
| Lebugle, 2017 | 1 | 1 | 0 | 0 | 0 | 0 | 0 | 1 | 0 | 1 | 4 | Moderate |
| Lenton et al., 1999 | 1 | 0 | 1 | 1 | 1 | 0 | 0 | 1 | 0 | 0 | 5 | Moderate |
| Loukaitou-Sideris et al., 2020 | 1 | 1 | 1 | 0 | 0 | 1 | 0 | 1 | 0 | 0 | 5 | Moderate |
| Loukaitou-Sideris et al., 2022 | 1 | 1 | 1 | 0 | 0 | 1 | 0 | 1 | 0 | 1 | 6 | Moderate |
| Macmillan et al., 2000 | 1 | 1 | 1 | 1 | 1 | 0 | 1 | 1 | 1 | 0 | 8 | High |
| Malik et al., 2020 | 1 | 0 | 1 | 0 | 1 | 0 | 0 | 0 | 0 | 0 | 3 | Low |
| Marmet et al., 2017 | 0 | 0 | 1 | 1 | 1 | 0 | 1 | 1 | 0 | 0 | 5 | Moderate |
| Mellgren et al., 2018 | 1 | 1 | 1 | 1 | 1 | 1 | 0 | 1 | 0 | 1 | 8 | High |
| Mishra et al. 2018 | 1 | 1 | 0 | 0 | 1 | 0 | 0 | 1 | 0 | 1 | 5 | Moderate |
| Mora et al., 2022 | 1 | 1 | 1 | 0 | 0 | 1 | 0 | 1 | 1 | 0 | 6 | Moderate |
| Moreno et al., 2022 | 1 | 1 | 1 | 0 | 1 | 0 | 0 | 1 | 1 | 0 | 6 | Moderate |
| Natarajan et al., 2017 | 1 | 0 | 0 | 0 | 0 | 1 | 0 | 1 | 0 | 1 | 4 | Moderate |
| Raj et al., 2021 | 1 | 0 | 1 | 1 | 1 | 0 | 0 | 1 | 0 | 1 | 6 | Moderate |
| Reed et al., 2019 | 1 | 1 | 1 | 0 | 0 | 1 | 0 | 1 | 1 | 1 | 7 | High |
| Saunders et al., 2017 | 1 | 1 | 1 | 0 | 0 | 1 | 1 | 1 | 1 | 0 | 7 | High |
| Shibata, 2020 | 1 | 0 | 1 | 0 | 1 | 0 | 0 | 1 | 0 | 1 | 5 | Moderate |
| Smith, 1994 | 1 | 1 | 1 | 0 | 1 | 0 | 0 | 1 | 0 | 1 | 6 | Moderate |
| Smith et al., 2022 | 1 | 1 | 1 | 1 | 1 | 0 | 0 | 1 | 0 | 1 | 7 | High |
| Solymosi et al., 2018 | 1 | 1 | 0 | 0 | 0 | 0 | 0 | 1 | 0 | 0 | 3 | Low |
| Whitfield et al., 2019 | 1 | 0 | 1 | 0 | 0 | 1 | 0 | 1 | 0 | 1 | 5 | Moderate |
