# Supplementary material for: Operationalizing Street Harassment Using Survey Instruments: A Systematic Review of Measuring Harassment in Public Spaces Using Surveys
Source: Trauma Violence Abuse. 2024 Feb 5;25(4):2609–21. doi: 10.1177/15248380231219258 (PMC11370194; doi:10.1177/15248380231219258)
Supplement: sj-docx-4-tva-10.1177_15248380231219258 – Supplemental material for Operationalizing Street Harassment Using Survey Instruments: A Systematic Review of Measuring Harassment in Public Spaces Using Surveys [file sj-docx-4-tva-10.1177_15248380231219258.docx]

| **First Author, Year** | **Location, Country** | **Sample type** | **Study design (sample size); sampling approach** | **Definition provided*** | **Delivery mode; measurement approach**** | **Measurement tool used** | **Tool/ scale used validated** | **Reporting period** | **Results disaggregated by sex** | **Prevalence estimates provided** | **Frequency of acts / experiences captured** | **Perpetrator details capture** |
| --- | --- | --- | --- | --- | --- | --- | --- | --- | --- | --- | --- | --- |
| Agrawal, 2020 | San Jose, California, USA | University students | Cross-Sectional Survey (n = 891); random | Yes | Online; List of behaviours or acts | The survey asked participants to indicate whether they had experienced a list of 16 acts across 3 contexts (travelling on, heading to, and waiting for the bus). Participants could also select “Other”. The behaviours were:   1. Using obscene/abusive language 2. Unwanted sexual looks or gestures 3. Sexual comments (about clothing, looks) 4. Whistling 5. Calling you babe, honey, sweetheart or something similar 6. Unwanted sexual teasing, remarks 7. Stalking (a stranger following you) 8. Indecent exposure 9. Making kissing sounds 10. Asking you questions of sexual nature 11. Groping, touching inappropriately 12. Showing pornographic images 13. Asked to have sex by a stranger 14. Masturbating in public 15. Pulling or playing with your hair 16. Unwanted kissing by a stranger 17. Other | No | Previous 3 years | Yes | Yes | No | No |
| Aguilar, 2018 | Mexico City, Mexico | Subway users | Cross-sectional survey (n = 5000); non-probability | No | Face-to-face / in person; List of behaviours or acts | New survey instrument developed from pilot surveys. Participants were asked “During your trip on line [COLOUR] did you experience any of the following”  There were 10 acts: 1. Shoving, elbowing 2. Physical fighting^ 3. Insults 4. Robbery^ 5. Unwanted Sexual Looks 6. Catcalls 7. Photos taken without consent 8. Groping without consent  9. Brushing against your body 10. Any other type of sexual violence: __ | No | During train trip | Yes | Yes | No | Yes, gender |
| Alam, 2010 | Bangladesh | Never-married adolescent girls aged 13 to 19 | Cross-sectional survey (n = 5106); random cluster probability | Yes | Face-to-face / in person; List of behaviours or acts | Survey undertaken as part of Bangladesh National Nutrition Programme. There were 6 questions asking Yes / No to behaviours on the way to school, college, work, or social visits.  1. Someone stared at you,  2. Blew whistle at you 3. Passed bad (sexual) comments or jokes about you 4. Enticed you 5. Threatened to kidnap you 6. Gave you bad (sex) proposals | No | Ever | N/A | Yes | No | Yes |
| Alvi, 2001 | Ontario, Canada | Women in public housing | Cross-sectional survey (n = 325); non-probability | No | Paper; List of behaviours or acts | Four modified versions of items from the Islington Crime Survey. Participants were asked to respond to four statements in relation to ‘harassment of women’, based on their experiences in the past 12 months, as follows:  “We realise that it may be difficult to discuss your experiences with crime in your neighbourhood. If we may, we would like to ask you a few questions about what happened to you in the last 12 months. Please choose your answer with a check mark. The response categories for the following questions are yes and no.”  In the last 12 months:   1. Did anyone ON THE STREET, IN A BAR OR OTHER PUBLIC PLACE ever insult you because they thought you were homosexual (gay or lesbian)? 2. Did anyone ON THE STREET, IN A BAR OR OTHER PUBLIC PLACE ever insult you because they did not like your skin color or religion? 3. Did anyone ON THE STREET, IN A BAR OR OTHER PUBLIC PLACE ever touch you sexually when you did not want to be touched (for example, your breasts, rear end, or genitals)? 4. Did anyone ON THE STREET, IN A BAR OR OTHER PUBLIC PLACE ever make sexual remarks about you or to you that made you feel uncomfortable? | Yes, Cronbach's alpha = .75 (for overall victimisation scale) | Previous 12 months | N/A | No | No | No |
| Anwar,  2019 | Islamabad, Lahore, Rawalpindi, Pakistan | Female university and college students | Cross-sectional survey (n = 543); non-probability | No | Online; List of behaviours or acts | A modified version of the Sexual Harassment Experience Questionnaire was used, with 19 items allocated to three sub-scales – physical (6 items, α = .78), verbal (5 items, α = .72), and non-verbal (8 items, α = .81) forms of sexual harassment. Participants were asked the following questions in relation to their experiences of being harassed in public and asked to rate them on a 5-point scale (never = 0, seldom = 1, sometimes = 2, often = 3, very often = 4).  Physical sexual harassment   1. Touched your hand while giving you something. 2. Stood close to you in a crowded place. 3. Collided with you while passing by. 4. Tried to have body touch with you while sitting. 5. Tried to kiss you against your will. 6. Tried to rape you.   Verbal sexual harassment   1. Passed unwanted comments on your appearance. 2. Said unwanted sexually oriented things to you. 3. Offered you an unwanted lift in a vehicle. 4. Threatened to spread rumours about you if you did not fulfil his sexual demands. 5. Threatened to harm you physically if you did not fulfil his sexual demands.   Nonverbal sexual harassment   1. Stared at you with dirty looks. 2. Not let you pass by. 3. Followed you in the street. 4. Whistled while looking at you. 5. Hummed filthy songs in your presence. 6. Tried to give you an unwanted card or gift. 7. Tried to give you a love letter you did not want. 8. Tried to undress himself in front of you.   Note. The article discusses results based on the location and perpetrator of sexual harassment, but does not provide details of how these questions are asked of participants. | Yes, physical harassment Cronbach's alpha = .78; Verbal harassment Cronbach's alpha = .72; Non-verbal harassment Cronbach's alpha = .81 | Ever | N/A | No | Yes | Yes |
| Anwar, 2020 | Finland | Female university students | Cross-sectional survey (n = 591); non-probability | No | Online, Paper; List of behaviours or acts | A modified version of the Sexual Harassment Experience Scale was used, with two items allocated to three sub-scales: physical (7 items, α = .89), verbal (6 items, α = .79), and non-verbal (7 items, α = .84) forms of sexual harassment.  Participants were asked “Has a man done any of the following things to you?” The acts were:  Physical Sexual Harassment   1. Touched you inappropriately while giving you something. 2. Tried to put his hand on yours, for example in computer teaching. 3. Tried to stand too close to you in a crowded place (eg. in an elevator). 4. Collided with you while passing by. 5. Tried to have body contact with you while sitting. 6. Tried to kiss you against your will. 7. Tried to rape you.   Verbal Sexual Harassment   1. Passed unwanted comments on your appearance with sexual allusions that you did not like. 2. Said unwanted sexually oriented things to you. 3. Offered you an unwanted lift in a vehicle. 4. Promised to promote you, or give you some other compensation, if you would agree to his sexual demands. 5. Threatened to spread false rumours about you if you did not fulfil his sexual demands. 6. Threatened to harm you physically if you did not fulfil his sexual demands.   Nonverbal Sexual Harassment   1. Stared at you with dirty looks. 2. Did not let you pass by. 3. Followed you in the street. 4. Whistled while looking at you. 5. Tried to give you an unwanted card or gift. 6. Tried to give you a love letter you did not want. 7. Tried to undress himself in front of you.   Note. The article discusses results based on the location and perpetrator of sexual harassment, but does not provide details of how these questions are asked of participants. | Yes, physical sexual harassment Cronbach's alpha = .89; Verbal sexual harassment Cronbach's alpha = .79; Non-verbal sexual harassment Cronbach's alpha = .84 | Ever | N/A | No | Yes | Yes |
| Anwar,  2020 | Ghana | Female university students and lecturers | Cross-sectional survey (n = 280); non-probability | No | Online, Paper; List of behaviours or acts | A modified version of the Sexual Harassment Experience Scale was shortened and adapted for use in Ghana. Participants were asked “Has a man done any of the following things to you?” about victimisation of sexual harassment in public and asked to rate them on a 5-point scale (never = 0, seldom = 1, sometimes = 2, often = 3, very often = 4).   1. Stared at you with dirty looks 2. Told dirty jokes in your presence 3. Shaked or pinched your palms 4. Tried to have bodily touch with you while sitting 5. Tried to stand too close to you in a crowded place 6. Followed you in the street 7. Offered you an unwanted lift in a vehicle 8. Tried to give you an unwanted card or gift 9. Tried to kiss you against your will 10. Tried to rape you 11. Tried to give or send you a text with sexual content 12. Passed unwanted comments on your appearance 13. Tried to undress himself in front of you 14. Threatened to spread rumours about you if you did not fulfil his sexual demands 15. Threatened to harm you physically if you did not fulfil his immoral sexual demands   Note. The article discusses results based on the location and perpetrator of sexual harassment, but does not provide details of how these questions are asked of participants. | Sexual Harassment: Cronbach's alpha = .86 | Ever | N/A | No | Yes | Yes |
| Awan, 2020 | Lahore, Pakistan | Female public transport commuters, 14-50 years old, from middle and low-middle income families | Cross-sectional survey (n = 205); no description given | No | Online; Direct query (e.g. ‘have you ever been sexually harassed in public? Y/N’); List of behaviours or acts | The survey included in the article asked participants “Have you ever faced harassment in public transport?” Responses were: Yes or No.  There was evidence of a list of behaviours being provided to participants (Table 3 in Awan, 2020). The behaviours included were:   1. Staring 2. Hooting (kissing noises & whistling 3. Stalking/Following 4. Pinching 5. Blocking the way 6. Rubbing 7. Sexual comment   Note. The author cites the scale administered in Anwar et al. (2019) but is unclear whether the entire scale is used to collect data. | No | Unspecified | N/A | Yes | Yes | No |
| Balsam, 2013 | USA | LGBT male, female, transgender and other gender adults | Cross-sectional survey (n = 900); non-probability | No | Online; List of behaviours or acts | Generated own survey items from previous study. There were 83 items across 10 factors. Only 1 factor was dedicated to discrimination/harassment which contained 4 items. The response categories were: 0 = did not happen to me/not applicable to me; 1 = it happened, and it bothered me NOT AT ALL; 2 = it happened, and it bothered me A LITTLE BIT; 3 = it happened, and it bothered me MODERATELY; 4 = it happened, and it bothered me QUITE A BIT; 5 = it happened, and it bothered me EXTREMELY.  The 4 items for discrimination/harassment were: 1. People staring at you when you are out in public because you are LGBT 2. Being verbally harassed by strangers because you are LGBT 3. People laughing at you or making jokes at your expense because you are LGBT 4. Being called names such as "fag" or "dyke" | Yes, factor loadings for each item onto "Discrimination/Harassment" 1. = .68 2. = .73 3. = .50 4. = .67 | Ever | No | No | No | No |
| Betts, 2019 | UK | 11-15 year old secondary school students | Cross-sectional survey (n = 118); non-probability | Yes | Online, Paper; List of behaviours or acts | Report contained 12 items which students could indicate they experienced. 1. Vehicle slowed, I was watched 2. Beeped at with a car horn 3. Cornered 4. Had bag taken/grabbed 5. Tripped up 6. Pushed/hit 7. Stared at 8. Talked about behind back 9. Whispered about 10. Called names 11. Laughed at 12. Other | No | As it occurred, period of 6-8 weeks | Yes | Yes | No | No |
| Brewster, 2019 | USA | Transgender people | Cross-sectional survey (n = 205); non-probability | No | Online; Direct query (e.g. 'have you ever been sexually harassed in public? Y/N'); List of behaviours or acts | The Interpersonal Sexual Objectification Scale (ISOS; Kozee et al., 2007) asks “How often have you experienced…” for the following 15 items: 1. Had someone whistling at you while walking down a street? 2. Had someone staring at your breasts/chest when you are talking to them? 3. Had someone evaluate your physical appearance? 4. Had someone stare at your body? 5. Had someone leer at your body? 6. Had someone make a rude, sexual remark about your body? 7. Had someone honk at you when you were walking down the street? 8. Had someone stare at one or more of your body parts? 9. Had someone make inappropriate sexual comments about your body? 10. Had someone gaze at your body or a body part, instead of listening to what you were saying? 11. Had someone make sexual comments or innuendos when noticing your body?  12. Had someone touch or fondle you against your will? 13. Had someone sexually harass you (on the job, in school, etc.)? 14. Had someone grab or pinch your private body areas against your will? 15. Had someone make a degrading sexual gesture towards you?  Note. The article did not include the items, and so these are derived from the original ISOS (Kozee et al., 2007). | Yes, Cronbach's alpha = .95 | Previous 12 months | No | No | Yes | No |
| Campos, 2017 | Mexico City, Mexico | Adult women who had reported physical or sexual IPV in the past year | Cross-sectional survey (n = 952); non-probability | No | Face-to-face / in person; List of behaviours or acts | Participants responded “Yes/No” to a list of behaviours comprising non-physical street harassment and physical street harassment. The reference period was the past month. The list of behaviours are derived from the results table (Table 1 in Campos et al., 2017). The non-physical street harassment behaviours were:   1. Made you feel uncomfortable with a whistling sound 2. Shouted or said obscene words to you 3. Looked at you in an uncomfortable way 4. Showed you some part of their body   The physical street harassment behaviours were:   1. Touched you with the hand without your consent 2. Touched you with some other part of the body without your consent 3. Pinched you 4. Was too close to you | No | Previous month | N/A | Yes | No | Yes |
| Carretta, 2020 | USA | Women 18-30 years old | Cross-sectional survey (n = 367); non-probability | Yes | Online; List of behaviours or acts | This adapted version of the Sexual Harassment Index (SHI; Fairchild & Rudman, 2008) removed the “Yes/No” items in favour of using only frequency questions. Response alternatives: 0 = never; 1 = once; 2 = once a month; 3 = 2-4 times pers month; 4 = every few days; 5 = every day. The questionnaire contained 9 items, presented as follows:  "In the past year, how frequently have you experienced…”   1. Catcalls, whistles, or stares from a stranger? 2. Unwanted sexual attention from a stranger? 3. Crude or offensive sexual jokes from a stranger? 4. Sexist remarks or behaviors from a stranger? 5. Seductive remarks or come ons from a stranger? 6. Unwanted touching or stroking from a stranger? 7. Subtle pressure to cooperate sexually from a stranger? 8. Direct pressure to cooperate sexually from a stranger? 9. Forceful fondling or grabbing from a stranger? | Yes, Cronbach's alpha = .92 Factor loadings ranged from .64 to .86 onto a single factor | Ever | N/A | No | Yes | No |
| Ceccato, 2021 | Australia, Brazil, Canada, China, Colombia, France, Italy, Japan, Mexico, Nigeria, Philippines, Portugal, Sweden, UK, USA | University students | Cross-sectional survey (n = 13323); non-probability | Yes | Online, Paper; List of behaviours or acts | There were 16 specific behaviours which were asked about across 3 transit environments (while in transit settings, on transit vehicles, to/from the transit stop or station).   The 16 items had 3 sub-scales.  Verbal Harassment:  1. Sexual comments (about body, clothing); 2. Kissing sounds;  3. Sexual names such as being called “babe,” “honey,” “sweetheart”;  4. Being asked for sex;  5. Sexual teas-ing;  6. Being asked questions of sexual nature;  7. Whistling;  8. Obscene/abusive language.   Physical Harassment:  9. Unwanted kissing; 10. Having one’s hair pulled/touched;  11. Being groped/touched inappropriately.   Non-Verbal/Non-Physical Harassment:  12. Unwanted sexual looks/gestures;  13. Masturbation in public; 14. Being shown porno-graphic images;  15. Indecent exposure;  16. Stalking | No | Previous 3 years | Yes | Yes | No | No |
| Ceccato, 2022 | Australia, Brazil, Canada, China, Colombia, France, Italy, Japan, Mexico, Nigeria, Philippines, Portugal, Sweden, UK, USA | University students | Cross-sectional survey (n = 13323); non-probability | Yes | Online, Paper; List of behaviours or acts | There were 16 specific behaviours which were asked about across 3 transit environments (while in transit settings, on transit vehicles, to/from the transit stop or station).   The 16 items had 3 sub-scales.  Verbal Harassment:  1. Sexual comments (about body, clothing); 2. Kissing sounds;  3. Sexual names such as being called “babe,” “honey,” “sweetheart”;  4. Being asked for sex;  5. Sexual teasing;  6. Being asked questions of sexual nature;  7. Whistling;  8. Obscene/abusive language.   Physical Harassment:  9. Unwanted kissing; 10. Having one’s hair pulled/touched;  11. Being groped/touched inappropriately.   Non-Verbal/Non-Physical Harassment:  12. Unwanted sexual looks/gestures;  13. Masturbation in public; 14. Being shown porno-graphic images;  15. Indecent exposure;  16. Stalking | No | Previous 3 years | Yes | Yes | No | No |
| Davidson, 2015 | USA | Female undergraduate university students | Cross-sectional survey (n = 501); non-probability | Yes | Online; List of behaviours or acts | The adapted Stranger Harassment Index (SHI; Fairchild & Rudman, 2008) contained 18 items. First, participants reported whether they had ever experienced nine different types of behaviors from strangers using a yes/no response. Participants then responded to the same nine behaviors rating the frequency of occurrence. The response alternatives were: 1 = once; 2 = once a month; 3 = 2-4 times pers month; 4 = every few days; 5 = every day. Stranger Harassment:   1. Have you ever experienced catcalls, whistles, or stares from a stranger? 2. Have you ever experienced unwanted sexual attention from a stranger? 3. Have you ever experienced crude or offensive sexual jokes from a stranger? 4. Have you ever experienced sexist remarks or behaviors from a stranger? 5. Have you ever experienced seductive remarks or come ons from a stranger? 6. Have you ever experienced unwanted touching or stroking from a stranger? 7. Have you ever experienced subtle pressure to cooperate sexually from a stranger? 8. Have you ever experienced direct pressure to cooperate sexually from a stranger? 9. Have you ever experienced forceful fondling or grabbing from a stranger? | Yes, Mean verbal harassment (Cronbach’s alpha = .78) subscale, mean sexual pressure (Cronbach’s alpha = .76) subscale, and mean total SHI (Cronbach’s alpha = .82) scores were calculated | Ever | N/A | No | Yes | No |
| Davidson, 2016 | USA | Female undergraduate university students | Cross-sectional survey (n = 501); non-probability | Yes | Online; List of behaviours or acts | The adapted Stranger Harassment Index (SHI; Fairchild & Rudman, 2008) contained 18 items. First, participants reported whether they had ever experienced nine different types of behaviors from strangers using a yes/no. Participants then responded to the same nine behaviors rating the frequency of occurrence. The response alternatives were: 1 = once; 2 = once a month; 3 = 2-4 times pers month; 4 = every few days; 5 = every day. Stranger Harassment:   1. Have you ever experienced catcalls, whistles, or stares from a stranger? 2. Have you ever experienced unwanted sexual attention from a stranger? 3. Have you ever experienced crude or offensive sexual jokes from a stranger? 4. Have you ever experienced sexist remarks or behaviors from a stranger? 5. Have you ever experienced seductive remarks or come ons from a stranger? 6. Have you ever experienced unwanted touching or stroking from a stranger? 7. Have you ever experienced subtle pressure to cooperate sexually from a stranger? 8. Have you ever experienced direct pressure to cooperate sexually from a stranger? 9. Have you ever experienced forceful fondling or grabbing from a stranger? | Yes, Cronbach's Alpha = .82 | Ever | N/A | Yes | Yes | No |
| del Mar Rodas-Zuleta, 2022 | Colombia | General population / community | Cross-sectional survey (n = 438); non-probability | Yes | Online, Paper; Direct query (e.g. 'have you ever been sexually harassed in public? Y/N'); List of behaviours or acts | Participants completed 1 question, asking whether they had experienced situations of gender-based violence (sexual and street harassment and sexual abuse) with a follow up asking for the specific behaviour. The behaviours were:   1. Compliments 2. Whistling 3. Sexual leering 4. Photos without permission 5. Touching/Groping 6. Intimidation 7. Stalking 8. Masturbation 9. Attempted Rape 10. Rape | No | Ever | Yes | Yes | No | No |
| DelGreco, 2020 | USA | Female university undergraduate students studying communication | Cross-sectional survey (n = 252); non-probability | Yes | Online; List of behaviours or acts | Questionnaire contained 28 items asking about the frequency of experiencing acts of street harassment. The response alternatives were: 0 = Never; 1 = Once in the past year; 2 = A few times in the past year; 3 = About once a month 4 = A few times a month; 5 = Almost every day; 6 = Multiple times a day.  1. A man whistled, yelled, or honked at you from his car while you were walking/waiting for the bus/riding a bike  2. A man blew you kisses or made other romantic gestures to you on the street  3. A man told you to smile  4. A man made negative comments about your appearance as you walked by (e.g., “keep the legs, lose the face”)  5. A man offered you money for sex when you were either walking or standing and waiting for someone  6. A man asked you for your name  7. A man told you how pretty or attractive you were as you walked down the street and then repeated these comments louder, trying to get your attention  8. A man slowed down his car so that he could drive beside you as you walked and either watched you or spoke to you  9. A man made sexually explicit gestures to you as you walked (e.g., pantomiming a blow job or grabbing his crotch)  10. A man complimented your appearance (e.g., “you have beautiful eyes,” “nice legs,” or “you’re beautiful”)  11. A man asked if you have a boyfriend or are married  12. A man commented on your weight saying that you are either too fat or too skinny  13. A man made sexual comments to you and then followed you as you walked  14. A man asked you for your phone number  15. A man yelled things like “hey sexy!” or “you’re fine!” from a car while driving past you as you were walking or waiting for someone  16. A man walked past you and commented on your weight, saying that he approves of your size  17. A man touched you as you walked past them (e.g., touching your waist, brushing a hand against your breast, or grabbing your hand)  18. A man called you insulting names as you walked past (e.g., “whore” or “bitch”)  19. A man approached the male person you were walking or sitting with and complimented him on your appearance or on his successful conquest of you  20. A man yelled comments about your appearance at you while you were jogging  21. A man walked past and directed non-verbal sounds at you (cat calls, wolf whistles, etc.)  22. A man stared at you in a sexual way as they walked past you on the street (e.g. leering or eyeing you up and down)  23. Construction workers yelled compliments to you about your appearance as you walked past their work site  24. A man made gestures and calls for you to come over to where he was standing  25. A man pulled his car over as you were walking and asked you to do sexually explicit things with him  26. A man called for your attention and when you ignored him begun shouting insults at you  27. A man showed you his penis on the street  28. A man aggressively touched you as you walked past him (e.g. slapping your buttocks, punching you, tripping you, or poking you) | Yes, Cronbach's alpha = .96 | Previous 12 months | N/A | No | Yes | No |
| DelGreco, 2021 | USA | Undergraduate university students | Cross-sectional survey (n = 348; 143 men, 205 women); non-probability | Yes | Online; List of behaviours or acts | Questionnaire contained 28 items asking about the frequency of experiencing acts of street harassment. The response alternatives were: 0 = Never; 1 = Once in the past year; 2 = A few times in the past year; 3 = About once a month 4 = A few times a month; 5 = Almost every day; 6 = Multiple times a day.  1. A man whistled, yelled, or honked at you from his car while you were walking/waiting for the bus/riding a bike  2. A man blew you kisses or made other romantic gestures to you on the street  3. A man told you to smile  4. A man made negative comments about your appearance as you walked by (e.g., “keep the legs, lose the face”)  5. A man offered you money for sex when you were either walking or standing and waiting for someone  6. A man asked you for your name  7. A man told you how pretty or attractive you were as you walked down the street and then repeated these comments louder, trying to get your attention  8. A man slowed down his car so that he could drive beside you as you walked and either watched you or spoke to you  9. A man made sexually explicit gestures to you as you walked (e.g., pantomiming a blow job or grabbing his crotch)  10. A man complimented your appearance (e.g., “you have beautiful eyes,” “nice legs,” or “you’re beautiful”)  11. A man asked if you have a boyfriend or are married  12. A man commented on your weight saying that you are either too fat or too skinny  13. A man made sexual comments to you and then followed you as you walked  14. A man asked you for your phone number  15. A man yelled things like “hey sexy!” or “you’re fine!” from a car while driving past you as you were walking or waiting for someone  16. A man walked past you and commented on your weight, saying that he approves of your size  17. A man touched you as you walked past them (e.g., touching your waist, brushing a hand against your breast, or grabbing your hand)  18. A man called you insulting names as you walked past (e.g., “whore” or “bitch”)  19. A man approached the male person you were walking or sitting with and complimented him on your appearance or on his successful conquest of you  20. A man yelled comments about your appearance at you while you were jogging  21. A man walked past and directed non-verbal sounds at you (cat calls, wolf whistles, etc.)  22. A man stared at you in a sexual way as they walked past you on the street (e.g. leering or eyeing you up and down)  23. Construction workers yelled compliments to you about your appearance as you walked past their work site  24. A man made gestures and calls for you to come over to where he was standing  25. A man pulled his car over as you were walking and asked you to do sexually explicit things with him  26. A man called for your attention and when you ignored him begun shouting insults at you  27. A man showed you his penis on the street  28. A man aggressively touched you as you walked past him (e.g. slapping your buttocks, punching you, tripping you, or poking you)  Note. The authors note that 27 items were asked of women, but it was not clear which item was left out, hence all are included above. | No | Ever | No | No | Yes | Yes |
| Doan, 2007 | USA | Transgender people | Cross-sectional survey (n = 149); non-probability | No | Not specified; Direct query (e.g. 'have you ever been sexually harassed in public? Y/N'); List of behaviours or acts | The questionnaire contained 1 item which asked ‘Have you ever felt threatened in your city in the last year?’ The response alternatives were: Hostile stares, hostile comments, physical harassment. | No | Previous 12 months | Yes | Yes | No | No |
| Emerson, 2016 | Northern Ireland, UK | General population / community | Cross-sectional survey (n = 5069); random probability | No | Face-to-face / in person; List of behaviours or acts | Understanding Society (Wave 3). There were 2 items asked about 7 different settings.  The items were: In the last 12 months, have you:  1. been called names, threatened, or shouted at, in any of these places? 2. been physically attacked in any of these places?  The places were: (a) on public transport (b) at or around a bus or train station (c) in commercial places like shopping centres, shops or petrol stations (d) in places of entertainment like theatres, cinema, cafes or restaurants (e) at pubs, nightclubs, discos or clubs (f) in car parks (g) outside, such as on the street, in parks or sports grounds | No | Previous 12 months | No | Yes | No | No |
| Fairchild, 2008 | USA | Female university students studying Introductory Psychology | Cross-sectional survey (n = 228); non-probability | Yes | Online; List of behaviours or acts | Adapted Sexual Experiences Questionnaire (SEQ; Fitzgerald et al., 1995) contained 18 items asking about the frequency of experiences. Participants first responded "Yes or no" to each item. If yes, they reported the frequency: 1 = once; 2 = once a month; 3 = 2-4 times pers month; 4 = every few days; 5 = every day   1. Have you ever experienced catcalls, whistles, or stares from a stranger? 2. Have you ever experienced unwanted sexual attention from a stranger? 3. Have you ever experienced crude or offensive sexual jokes from a stranger? 4. Have you ever experienced sexist remarks or behaviors from a stranger? 5. Have you ever experienced seductive remarks or come ons from a stranger? 6. Have you ever experienced unwanted touching or stroking from a stranger? 7. Have you ever experienced subtle pressure to cooperate sexually from a stranger? 8. Have you ever experienced direct pressure to cooperate sexually from a stranger? 9. Have you ever experienced forceful fondling or grabbing from a stranger? | Yes, factor analysis. Two-factor solution, all factor loadings above .50. Overall Cronbach’s alpha = .85 Verbal subscale Cronbach’s alpha = .83 Sexual pressure Cronbach’s alpha = .75 | Ever | N/A | Yes | Yes | No |
| Fairchild, 2010 | Unspecified (web survey) | Women | Study 1: Cross-sectional survey (n = 1277); non-probability | Yes | Online; List of behaviours or acts | Adapted Sexual Experiences Questionnaire (SEQ; Fitzgerald et al.1995) modified by Fairchild and Rudman (2008). Questionnaire contains 9 behaviours. If participants select "yes" they are asked for the frequency of this experience. The response alternatives were: 1 = once; 2 = once a month; 3 = 2-4 times pers month; 4 = every few days; 5 = every day  Stranger Harassment (yes/No):   1. Have you ever experienced catcalls, whistles, or stares from a stranger? 2. Have you ever experienced unwanted sexual attention from a stranger? 3. Have you ever experienced crude or offensive sexual jokes from a stranger? 4. Have you ever experienced sexist remarks or behaviors from a stranger? 5. Have you ever experienced seductive remarks or ‘come ons’ from a stranger? 6. Have you ever experienced unwanted touching or stroking from a stranger? 7. Have you ever experienced subtle pressure to cooperate sexually from a stranger? 8. Have you ever experienced direct pressure to cooperate sexually from a stranger? 9. Have you ever experienced forceful fondling or grabbing from a stranger? | No | Ever | N/A | Yes | Yes | No |
| Ferrer-Perez, 2021 | Spain | 18 and 30 year olds | Cross-sectional survey (n = 538); non-probability | Yes | Online; Direct query (e.g. 'have you ever been sexually harassed in public? Y/N') | Based on the available information, there were 4 items relevant to street harassment. Participants were asked to indicate whether they had experienced or witnessed "Sexual harassment" and "Street sexual harassment". The response alternatives reported by the authors were: 1 = No, never; 2 = Yes, once; 3 = Yes, more than once; 4 = Yes, regularly | No | Ever | Yes | Yes | Yes | No |
| Fileborn, 2019 | Melbourne, Australia | Adults who had experienced street harassment (self-defined)g | Cross-sectional survey (n = 292); non-probability | Yes | Online; Direct query (e.g. 'have you ever been sexually harassed in public? Y/N'); List of behaviours or acts | Author created items. All items for this section are included since the order is important (e.g., Yes / No occurs before types are outlined).   1. "Have you ever experienced street harassment in Melbourne?" (Yes / No) 2. On average, how frequently do you experience street harassment in Melbourne?    1. Daily (4 or more times per week)    2. Weekly (3 or less times per week)    3. A few times a month    4. A few times a year    5. Other (please specify) 3. When did you most recently experience street harassment in Melbourne? 4. Which forms of street harassment have you EVER experienced? (select all that apply)    1. Excessive staring/leering    2. Verbal comments and abuse    3. Prolonged unwanted conversation    4. Unwanted sexual propositions    5. Wolf-whistling    6. Honking car horn    7. Following    8. Kissing    9. Groping    10. Had objects thrown at you    11. Had someone rub up against you    12. Other (please specify) 5. Which forms of street harassment do you MOST COMMONLY experience? (select all that apply)    1. Excessive staring/leering    2. Verbal comments and abuse    3. Prolonged unwanted conversation    4. Unwanted sexual propositions    5. Wolf-whistling    6. Honking car horn    7. Following    8. Kissing    9. Groping    10. Had objects thrown at you    11. Had someone rub up against you    12. Other (please specify) 6. How old were you when you first experienced street harassment? 7. Have your experiences of street harassment changed over time? If so, how? 8. In which locations do you MOST COMMONLY experience street harassment? 9. What time of day do you MOST COMMONLY experience street harassment? 10. Have you ever been targeted for street harassment on the basis of your: (select all that apply)   Qualitative items asking to elaborate on what the typical experience is like, what the most recent experience was like, and what the most significant or harmful experiences were. | No | Ever | No | No | Yes | Yes |
| Gurrola-Peña, 2022 | Mexico | 15-30 year olds | Cross-sectional survey (n = 250 - internal consistency), Cross-sectional survey (n = 400 - confirmatory factor analysis); non-probability | Yes | Online; List of behaviours or acts | Mexican Sexual Street Harassment Questionnaire. There were originally 20 items with five response options ranging from "It has never happened to me" to "It has happened to me very frequently in the last year"  There were 17 items retained from the exploratory factor analysis were: 1. Someone whistles at you 2. Someone stares at an intimate part of your body 3. Someone blows kisses at you 4. Someone honks the car horn to address you 5. Someone catcalls you 6. Someone makes vulgar gestures towards you 7. Someone tells you offensive sexual words or phrases 8. Someone tells you words or phrases that refer to parts of your body 9. Someone touches non-intimate parts of your body 10. Someone presses their genitals against your body 11. Someone touches an intimate part of your body 12. Someone gets unnecessarily close to you 13. Someone blocks your way while you walk 14. Someone chases you down 15. Someone intentionally brushes against your genitals 16. Someone masturbates in front of you 17. You witness exhibitionist acts directed at you | Yes, EFA identified three latent factors: non-physical sexual harassment (8 items; a = .89); physical sexual harassment (7 items; Cronbach's alpha .81); explicit sexual harassment (3 items; Cronbach's alpha .73). CFA was performed with the data using a larger sample size. It was a hierarchical model where each of the latent factors loaded onto a second-order latent factor "Street Sexual Harassment." All factor loadings were above .50 and the model demonstrated acceptable fit across approximate fit indices: GFI = .97; RMR = .46; RMSEA = .06; CFI = .95; NFI = .93; TLI = .94. | Unspecified | No | No | Yes | No |
| Heesch, 2011 | Queensland, Australia | Cyclists | Cross-sectional survey (n = 1830); non-probability | No | Online; Direct query (e.g. 'have you ever been sexually harassed in public? Y/N'); List of behaviours or acts | Survey asked one gateway question with five possible follow-up items “While cycling have you perceived any intentional harassment from motorists or their passengers in the previous 12 months?” Respondents who answered yes were then asked whether they viewed it as:   1. Deliberately driving too close 2. Tailgating (causing fear / anxiety) 3. Throwing objects, deliberately 4. Blocking their path, shouting abuse 5. Making obscene gestures / sexual harassment | No | Previous 12 months | Yes | Yes | No | No |
| Imtiaz, 2021 | Islamabad; Karachi; Lahore, Pakistan | Victims of sexual harassment who had experienced sexual harassment within the past 6 months | Cross-sectional survey (n = 452); non-probability | Yes | Online; List of behaviours or acts | Adapted Sexual Harassment Experience Questionnaire (SHEQ; Tariq and Kamal, 1997) for public spaces with one gateway question. The gateway question was an author-created item: “In the past six months, at what public places have you been harassed?” The response alternatives were: parks, universities, shopping centers, streets, and transport areas, other than public places.  The SHEQ usually contains 35 items, but 33 items were used since 2 items related to workplace harassment. The SHEQ has 3 subscales: Gender Harassment, Unwanted Sexual Attention, and Sexual Coercion. The response alternatives were not explicitly mentioned in the article, but the range for each subscale was 1*number of items to 4*number of items, suggesting it was the 4-point frequency from Tariq and Kamal (1997): 1 = Never; 2 = Once; 3 = A few times; 4 = Very frequent.  Gender Harassment:   1. Dirty jokes 2. Starring (sic.) 3. Admired your dress 4. Show pornographic material 5. Humming filthy songs 6. Admired your face or hair 7. Tried to talk about vulgar movie or program   Unwanted Sexual Attention:   1. Appreciated your body/figure 2. Tried to make you sit with him/her Invited for an outing or going to a restaurant with him/her to eat 3. Flirt 4. Offered lift in a car 5. Tried to give you a card 6. Sexting/flirting on call 7. Took interest in your personal life 8. Talk about sexual life 9. Tried to probe your sexual life 10. Collided with you while passing by 11. Touch your hand intentionally while giving something 12. Calling you darling, sweetheart, etc. 13. Put his hand on your shoulder or back 14. Love letter 15. Body touch while working 16. Bad talk/sexual talk 17. Pat on your shoulder while praising 18. Tried to kiss you 19. Tried to rape you   Sexual Coercion   1. Delayed your work so that you go to him/her again and again 2. Assured you some benefit if you could fulfill immoral demands 3. Threatened you for romantic ties 4. Tried to defame you for not fulfilling his/her demands 5. Forced you for immoral demands by exploiting your hardships 6. Threatened you for physical/sexual relationship | Yes, gender harassment Cronbach's alpha = 0.64  Unwanted sexual attention Cronbach's alpha = 0.83  Sexual coercion Cronbach's alpha = 0.76 | Not specified but sampling frame within past 6 months | Yes | Yes | Yes | Yes, gender |
| Infante-Vargas, 2022 | Saltillo, Coahuila, Mexico | Female public transport users | Cross-sectional survey (n = 611); non-probability | Yes | Online; Direct query (e.g. 'have you ever been sexually harassed in public? Y/N'); List of behaviours or acts | ‘Have you experienced any of these behaviours in the *last year* of using the public transport of Saltillo?; ‘Have you experienced any of these behaviours *since you started* using the public transport of Saltillo?’  14 behaviours listed for both questions (yes / no):  1. Catcalling or unwanted sexual remarks  2. Insults or despective words about you or other women  3. Unsolicited ear whispering or whistling  4. Lascivious looks  5. Unwanted touching in any part of the body  6. The perpetrator(s) showed their genitals  7. The perpetrator(s) touched their own genitals or masturbated while in front of you  8. Unsolicited spanking  9. Unwanted groping with sexual intentions  10. The perpetrator(s) took pictures of videos of you without your consent  11. The perpetrator(s) made sexual propositions to you or other women  12. The behaviour of the perpetrator(s) made you feel unsafe or in threat of a sexual attack  13. The perpetrator(s) chased you with the intention to sexually harass or attack  14. The perpetrator(s) forced you to perform sexual acts | No | Previous 12 months; Ever | N/A | Yes | Yes | No |
| Jabeen, 2017 | Lahore, Pakistan | Women 15- 35 years old | Cross-sectional survey (n = 600); non-probability | Yes | Face-to-face / in person; Direct query (e.g. 'have you ever been sexually harassed in public? Y/N'); List of behaviours or acts | Based on the table of results, the survey contained three items asking about harassment.  1. Facing incident/s of harassment in a day - Once - Twice - Thrice or more  2. Type of harassment by conductors and drivers of public transport - Touching - Fixing view mirror - Delay tactics - Meaningful songs  3. Forms of Harassment - Stare - Touch - Cat calls - Chase - Meaningful songs - Sexual gestures | No | Ever | N/A | Yes | Yes | Yes, occupation, age, gender |
| Kash, 2019 | Colombia and Bolivia | Female public transport users | Cross-sectional survey (n = 255); stratified random cluster probability | Yes | Face-to-face interviews; List of behaviours or acts | There were two items pertinent to sexual harassment asked only to women. Women were asked “Has this ever happened to you?” (Yes, no)   1. Someone touched you inappropriately or assaulted you in the bus or station 2. Someone touched you in a way that you feel uncomfortable, but you didn’t know if it was on purpose (Colombia sample only)   Participants who responded yes were asked how many times they had experienced the harassment.  Note. The authors conceptualise the first item as “Sexual assault”. | No | Ever | N/A | Yes | Yes | No |
| Kearl, 2014 | USA | General population / community | Cross-sectional survey (n = 2000); stratified probability | Yes | Online; List of behaviours or acts | Please indicate if you have ever experienced unwanted verbal behaviors in public spaces, by a person you did not know. This includes experiences you had when you were younger. (Select all that apply).   1. Someone whistling at you, going “Psssst,” or saying things like, “Hey Baby,” “Mmmm Sexy,” “Yo Shorty,” “Mami,” “Mamacita,” “Papi,” “Give me a smile,” or similar comments 2. Someone talking about your body parts inappropriately or offensively (such as your legs, crotch, butt, or breasts) or making obscene sounds 3. Someone calling you a sexually explicit name or saying a sexually explicit comment to or about you (“I want to do BLANK to you,” or “I want you to do BLANK to me”) 4. Someone calling you a homophobic or transphobic slur, like “Fag,” “Dyke,” or “Tranny” 5. Someone calling you a sexist slur, like “Bitch,” “Slut,” or “Ho” 6. None   Please indicate if you have ever experienced unwanted physically aggressive behaviors in public spaces, by a person you did not know. This includes experiences you had when you were younger. (Select all that apply.)   1. Someone flashing or exposing his/her genitals to you 2. Someone following you without your permission 3. Someone purposely touching you or brushing up against you in an unwelcome, sexual way 4. Someone forcing you to do something sexual without your permission 5. None   Where have you encountered harassment in a public space by a stranger? (Select all that apply.)   1. While on the street or sidewalk, either on foot (such as walking, standing, sitting, or jogging) or on a bicycle or skateboard 2. In a car, truck, van, taxi, or motorcycle (as the driver or as a passenger) 3. On public transportation like a bus, trolley, train, subway, or airplane 4. In a store, restaurant, movie theater, or mall 5. At a sports arena, gym, ball field, park or beach 6. Other   In your experience/s who were the harassers? (Select all that apply.)   1. One male 2. Two or more males 3. One female 4. Two or more females 5. A group of males and females 6. Other   On average, approximately how often have you encountered harassment in public spaces by strangers across your lifetime? (Select only one response.)   1. Once 2. Rarely 3. Sometimes 4. Often 5. Daily | No | Ever | Yes | Yes | Yes | Yes, gender, number of perpetrators |
| Khairat, 2016 | Giza Pyramids area, Sharm-El Sheikh, and Hurghada, Egypt | Tourists in Giza Pyramids area, Sharm-El Sheikh, and Hurghada | Cross-sectional survey (n = 218); non-probability | Yes | Telephone; Direct query (e.g. 'have you ever been sexually harassed in public? Y/N'); List of behaviours or acts | Questionnaire contained four items.   1. Participants were asked "Have you ever experienced harassment?" The response alternatives were: yes; no. 2. Participants were then asked how many times they had experienced harassment.    1. Once    2. Twice    3. Thrice 3. Participants were asked about which ways they had been harassed. Options included:    1. Robbery    2. Assault    3. Rape    4. Abuse    5. Fraud. 4. Participants were also asked about types of harassment. Options were:    1. Verbal harassment    2. Non-verbal harassment    3. Physical harassment    4. Begging    5. Drug peddling    6. Pestering   Note. The author conceptualises question 3 as harassment. | No | While on holiday | Yes | Yes | Yes | Yes, occupation |
| Lebugle, 2017 | France | General population / community | Cross-sectional survey (n = 27268); non-probability | Yes | Telephone; List of behaviours or acts | VIRAGE Survey (2015). Participants answered 11 questions. The response alternatives were: yes; no.   1. Have you been whistled at, called or approached under the pretext of chatting up? 2. Have you been insulted in the street, in public transport or at a public place near your home, for example? 3. Have you been persistently sexual advances despite your refusal? 4. Have you been subject to persistent sexual advances despite your refusal? 5. Have you fought, or exchanged punches with one or more persons during a street fight? 6. Have you been slapped, shaken, punched or physically assaulted in another way in a public space? 7. Have you been threatened with a weapon, has someone tried to strangle you, to endanger your life or kill you? 8. Have you been approached by an exhibitionist or a voyeur in a public space? 9. Has anyone, against your will, touched your breast or your buttocks, cornered you in order to kiss you, rubbed or pressed against you? [for men]: Has anyone against your will rubbed or pressed against you? 10. Has someone forced you to perform or submit to sexual touching, has someone attempted to have sexual intercourse with you against your will, or succeeded in doing so? 11. Has someone ever forced you to engage in other sexual acts or practices? | No | Previous 12 months | Yes | Yes | Yes | Yes, relation to victim, whether same perpetrator over multiple incidents |
| Lenton, 1999 | Canada | Women 18-65 years old | Cross-sectional survey (n = 1990); two-stage probability | No | Online; List of behaviours or acts | Participants were asked about five different experiences since they were 16 years of age. The response alternatives were: never, once, more than once. 1. A man stared at you in a way that made you feel uncomfortable 2. A man shouted unwanted sexual comments at you 3. A man indecently exposed himself to you 4. A man followed you on foot or in a vehicle 5. A man touched or tried to touch you in a sexual way  Participants could describe "Other" experiences. | No | Ever | Yes | Yes | Yes | Yes, asked if man had perpetrated act |
| Loukaitou-Sideris, 2020 | Los Angeles, USA | University students | Cross-sectional survey (n = 1284); non-probability | Yes | Online, Paper; List of behaviours or acts | There were 16 specific behaviours which were asked about across 3 transit environments (while in transit settings, on transit vehicles, to/from the transit stop or station).   The 16 items had 3 sub-scales.  Verbal Harassment:  1. Sexual comments (about body, clothing); 2. Kissing sounds;  3. Sexual names such as being called “babe,” “honey,” “sweetheart”;  4. Being asked for sex;  5. Sexual teasing;  6. Being asked questions of sexual nature;  7. Whistling;  8. Obscene/abusive language.   Physical Harassment:  9. Unwanted kissing; 10. Having one’s hair pulled/touched;  11. Being groped/touched inappropriately.   Non-Verbal/Non-Physical Harassment:  12. Unwanted sexual looks/gestures;  13. Masturbation in public; 14. Being shown porno-graphic images;  15. Indecent exposure;  16. Stalking | No | Previous 3 years | Yes | Yes | No | No |
| Loukaitou-Sideris, 2022 | Australia, Brazil, Canada, China, Columbia, France, Italy, Japan, Mexico, Nigeria, Philippines, Portugal, Sweden, United Kingdom, USA | University students | Cross-sectional survey (n = 11710); non-probability | Yes | Telephone; List of behaviours or acts | There were 16 specific behaviours which were asked about across 3 transit environments (while in transit settings, on transit vehicles, to/from the transit stop or station).   The 16 items had 3 sub-scales.  Verbal Harassment:  1. Sexual comments (about body, clothing); 2. Kissing sounds;  3. Sexual names such as being called “babe,” “honey,” “sweetheart”;  4. Being asked for sex;  5. Sexual teasing;  6. Being asked questions of sexual nature;  7. Whistling;  8. Obscene/abusive language.   Physical Harassment:  9. Unwanted kissing; 10. Having one’s hair pulled/touched;  11. Being groped/touched inappropriately.   Non-Verbal/Non-Physical Harassment:  12. Unwanted sexual looks/gestures;  13. Masturbation in public; 14. Being shown porno-graphic images;  15. Indecent exposure;  16. Stalking | No | Previous 3 years | Yes | Yes | No | No |
| Macmillan, 2000 | Canada | Women living in Canada | Cross-sectional survey (n = 12300); stratified probability | Yes | Face-to-face / in person; List of behaviours or acts | There were four items which measured experiences of stranger sexual harassment. Participants were asked whether they had ever experienced:   1. Received an obscene phone call 2. Received unwanted attention (i.e., anything that does not involve touching, such as cat calls, whistling, leering, or blowing kisses) 3. Been followed in a manner that frightened them 4. Indecent exposure | No | Ever | N/A | Yes | No | No |
| Malik, 2020 | Lahore, Pakistan | Female bus rapid transit users | Cross-sectional survey (n = 429); random probability | No | Telephone; Direct query (e.g. 'have you ever been sexually harassed in public? Y/N'); Categories of behaviours (e.g. verbal, non-verbal, physical, sexual) | There were four survey items. The first two asked participants whether they had experienced harassment. The response alternatives were: 1 = Highly agree through to 5 = Highly disagree.   1. Have you experienced harassment in BRT buses? 2. Have you experienced harassment in BRT stations?   Two follow-up questions asked about the type of harassment. The response alternatives were: Nothing too disturbing; verbal; physical; visual; other   1. Type of harassment faced in BRT buses? 2. Type of harassment faced in BRT stations? | No | Ever | N/A | No | No | No |
| Marmet, 2017 | Switzerland | General population / community | Cross-sectional survey (n = 2474, unweighted; n = 2469, weighted); two-stage random probability | No | Paper; Direct query (e.g. 'have you ever been sexually harassed in public? Y/N'); List of behaviours or acts | The questionnaire asked participants six items in total. There were four items which formed a scale.   1. Has someone who had been drinking harassed or bothered you on the street or in some other public space? 2. Has someone who had been drinking made you afraid when you encountered them on the street? 3. Have you been kept awake at night by drunken noise?^ 4. Have you felt unsafe in a public place because of someone's drinking?^  ^ Denotes items that don't seem to measure harassment, but are included on the scale by authors.   There were two additional items measuring "Verbal and physical aggression outside of relationships"   1. Has someone who had been drinking called you names or otherwise insulted you? 2. Has someone who had been drinking harmed you physically? | No | Previous 12 months | No | Yes | Yes | Yes, relation to victim |
| Mellgren, 2018 | Malmo, Sweden | Female university students at Malmo University | Cross-sectional survey (n = 1941); non-probability | Yes | List of behaviours or acts | A single-item measure was used. “Have you been exposed to any unwanted sexual contact such as touching, kissing, grabbing, or fondling during the past 12 months?” The response alternatives were: yes; no. | No | Previous 12 months | N/A | Yes | Yes | Yes, relation to victim |
| Mishra, 2018 | Kathmandu, Nepal | Female health science students studying in Manmohan Memorial Institute of Health Sciences | Cross-sectional survey (n = 396); stratified random probability | Yes | Online; Direct query (e.g. 'have you ever been sexually harassed in public? Y/N');  Categories of behaviours (e.g. verbal, non-verbal, physical, sexual) | Two items were used. The first item asked whether participants had “Ever been sexually harassed in a public vehicle?” The response alternatives were: yes; no.  The second item asked what type of sexual harassment was experienced. The response alternatives, which were not mutually exclusive, were:  verbal; non-verbal; physical. | No | Previous 6 months | N/A | Yes | Yes | Yes, occupation, age |
| Mora, 2022 | Chicago, Detroit, USA | Adolescents at a Chicago and a Detroit high school | Cross-sectional survey (n = 416); non-probability | Yes | Face-to-face / in person; List of behaviours or acts | Adapted version of the Sexual Experiences Questionnaire Latina (SEQL; Cortina,2001). The survey included 6 items, with response alternatives including frequencies from 1 = never to 5 = most of the time.   1. Made to feel uncomfortable by someone staring at you 2. Someone whistle at you or make kissing noises at you 3. Someone slowly look at your entire body 4. Someone say offensive or embarrassing things about your body 5. Someone give you a gender-based look that made you uncomfortable 6. Someone call you an inappropriate name, like "Mamacita" or "Marica" | Yes, Previous studies. Also, Alpha for entire sample, boys, and girls, respectively: .93, .94, and .91 | Previous 12 months | No | Yes | Yes | No |
| Moreno, 2022 | Austria, Belgium, Bulgaria, Croatia, Cyprus, Czech Republic, Denmark, Estonia, Finland, France, Germany, Greece, Hungary, Ireland, Italy, Latvia, Lithuania, Luxembourg, Malta, Netherlands, Poland, Portugal, Romania, Slovakia, Slovenia, Spain, Sweden, United Kingdom | Adult women | Cross-sectional survey (n = 41895); random probability | Yes | List of behaviours or acts | The survey instrument asked eleven questions in total. First, participants were asked: “Since you were 15 years old until now, has the same person repeatedly done one or more of the following things to you?” The possible answers were: No; Yes, has this happened 2 - 5 times; or Yes, 6 or more times. The acts were:   1. Loitered or waited for you outside your home, workplace or school without a legitimate reason; 2. Deliberately followed you around   Then, participants were asked if they had ever experienced nine different acts. Three occurred in a virtual space, and were not relevant for our review.  Physical (relevant to this review):   1. Suffered unwelcome touching, hugs, or kisses 2. Been leered or stared at in a way that made you feel intimidated 3. Received sexually suggestive comments or told jokes that made you feel offended 4. Received inappropriate invitations to appointments 5. Suffered indiscreet questions about privacy that are offensive 6. Received indiscreet comments about physical appearance that are offensive   Virtual (not relevant for this review):   1. Received or have been shown sexually explicit illustrations or pictures that are offensive 2. Received unwanted and sexually explicit e-mails or MSMs that are offensive 3. Suffered inappropriate and offensive approaches on social networks or websites such as Facebook or in chats. | Yes, Factor analysis undertaken to examine construct validity. Cronbach's alpha for physical sexual harassment subscale = 0.83 | Ever (since 15 yrs of age) | N/A | Yes | No | No |
| Natarajan, 2017 | New York City, USA | Female university student | Cross-sectional survey (n = 147); non-probability | No | Online; List of behaviours or acts; Direct query (e.g. 'have you ever been sexually harassed in public? Y/N'); Categories of behaviours (e.g. verbal, non-verbal, physical, sexual) | A questionnaire containing three items. The first asked “Have you experienced or witnessed any of the following types of sexual harassment on your commute to/from John Jay? Select all that apply” 1. A stranger following you 2. Whistling 3. Cat-calls 4. Inappropriate comments 5. Inappropriate touching 6. Groping 7. Staring 8. Indecent exposure 9. Your picture taken without your permission  "Since attending John Jay College, about how many times on average have you experienced sexual harassment on your commute to/from John Jay?" 1. Everyday 2. More than 4 times a week 3. 2-3 times a week 4. Once a week 5. 2-3 times a month 6. Once a month 7. Less than once a month 8. Never  "Have you experienced or witnessed any of the following crimes on your commute to/from John Jay? Select all that apply" Only one option was harassment. - Sexual Harassment | No | Ever | N/A | Yes | Yes | No |
| Raj, 2021 | USA | General population / community | Cross-sectional survey (n = 2009); stratified probability | No | Paper; List of behaviours or acts | The Stop Street Harassment survey (2018). Participants were asked about 15 behaviours, 11 of which were harassment in the street. The behaviours were presented to participants in a matrix containing 16 different locations. The behaviours were:  Physical 1. Someone purposely touching you or brushing up against you in an unwelcome, sexual way.  2. Someone physically following you without your permission 3. Someone flashing or exposing their genitals to you without your permission  Verbal 1. Someone repeatedly asking you for a date or your phone number when you've said no or ignored them. 2. Someone saying you must date them or do a sexual act for them in exchange for something (such as a good grade, a promotion, a job, drugs, food, or something) 3. Someone making threats to harm you, to harm someone you know, or to share personal information you don't want shared (such as your sexual orientation) 4. Someone talking about your body parts inappropriately or offensively (such as your legs, crotch, butt, or breasts), saying sexually explicit comments (“I want to do BLANK to you”) or asking inappropriate sexual questions 5. Someone misgendering you or calling you a homophobic or transphobic slur, like “Fag,” “Dyke,” or “Tranny.” 6. Someone calling you a sexist slur, like “Bitch,” “Slut,” “Cunt,” “Ho” or “Thot.”  7. Someone saying things like, “Hey Baby,” “Mmmm Sexy,” “Yo Shorty,” “Mami/Mamacita,” “Give me a smile,” or similar comments in a way that is disrespectful and/or unwanted and/or made you feel unsafe.  8. Someone whistling, honking, making kissy noises, “Pssst” sounds, or leering/staring aggressively at you. | No | Ever | Yes | Yes | No | No |
| Reed, 2019 | California-Mexico border, USA | Adolescent girls 15--19 at an adolescent health clinic | Cross-sectional survey (n = 159); non-probability | Yes | List of behaviours or acts | Sexual harassment victimisation was assessed using items from the American Association of University Women (AAUW) survey (AAUW, 2001). These 4 items asked participants whether a man/boy or group of men/boys have, in the past 6 months (a = .56):   1. Made unwanted sexual comments, jokes, or gestures towards you in public; 2. Exposed themselves sexually in public; 3. Touched, grabbed, or pinched them in a sexual way that you did not want 4. Touched you with any part of their body including getting too close or rubbing up against them when you did not want this.   There were two additional items which asked where the harassment occurred and who perpetrated it.  Participants were asked “In the past, when boys or men have done any of these things to you, where did it happen?” The responses were: at school; at home; at work; on public transportation; my neighbourhood; another neighbourhood nearby; other place. Responses were not mutually exclusive.    “When boys or men have done any of these things to you, who did this?” The responses were: boys/men I did not know; boys/men I knew of or have seen but am not friendly with; boys/men I knew pretty well or who are my friends. Responses were not mutually exclusive. | Yes, Cronbach's alpha .56 | Previous 6 months | N/A | Yes | Yes | Yes, relation to victim |
| Saunders, 2017 | USA | Female undergraduate university students | Cross-sectional survey (n - 143); convenience non-probability | Yes | Online; List of behaviours or acts | The adapted Sexual Experiences Questionnaire (Fairchild & Rudman, 2008) contained nine items describing different behaviours. Participants report how often they experienced each behaviour on a scale of 1 (never) to 7 (everyday). The items were as follows:  In thinking about your experiences out in public, have you ever... 1. Experienced catcalls, whistles, or stares from a stranger? 2. Experienced unwanted sexual attention or interaction from a stranger? 3. Experienced crude or offensive sexual jokes from a stranger? 4. Experienced sexist remarks of behaviors from a stranger? 5. Experienced seductive remarks or "come ons" from a stranger? 6. Experienced unwanted touching or stroking from a stranger? 7. Experienced subtle pressure to cooperate sexually from a stranger? 8. Experienced direct or explicit pressure to cooperate sexually from a stranger? 9. Experienced direct or forceful fondling or grabbing from a stranger? | Yes, Cronbach's alpha reported - 0.91 | Ever | N/A | Yes | Yes | No |
| Shibata, 2020 | Tokyo, Japan | College students | Cross-sectional survey (n = 400); random quota probability | No | Online; Direct query (e.g. 'have you ever been sexually harassed in public? Y/N') | Participants were asked whether they had experienced groping (yes / no) on either the railway or bus. | No | Previous 3 years | Yes | Yes | No | No |
| Smith, 1994 | Toronto, Canada | General population / community | Cross-sectional survey (n = 604); random probability | Yes | Direct query (e.g. 'have you ever been sexually harassed in public? Y/N') | A single-item measure was used. “For this survey, abuse means being pushed, grabbed, slapped, punched, kicked, beaten up, attacked with a weapon, or physically attacked in any other way. Since you were 16 years of age, has any male stranger abused you, or tried to abuse you, in public?" The response alternatives were: yes; no. | No | Ever (since 16 yrs of age) | No | Yes | Yes | Yes, relation to victim |
| Smith, 2022 | USA | General population / community | Cross-sectional survey (n = 2205); random probability | Yes | Online; List of behaviours or acts | The Stop Street Harassment survey (2019). Participants were asked about 15 behaviours, 11 of which were harassment in the street. The behaviours were presented to participants in a matrix containing 15 different locations. The behaviours were:  Physical 1. Someone purposely touching you or brushing up against you in an unwelcome, sexual way.  2. Someone physically following you without your permission 3. Someone flashing or exposing their genitals to you without your permission  Verbal 1. Someone repeatedly asking you for a date or your phone number when you've said no or ignored them. 2. Someone saying you must date them or do a sexual act for them in exchange for something (such as a good grade, a promotion, a job, drugs, food, or something) 3. Someone making threats to harm you, to harm someone you know, or to share personal information you don't want shared (such as your sexual orientation) 4. Someone talking about your body parts inappropriately or offensively (such as your legs, crotch, butt, or breasts), saying sexually explicit comments (“I want to do BLANK to you”) or asking inappropriate sexual questions 5. Someone misgendering you or calling you a homophobic or transphobic slur, like “Fag,” “Dyke,” or “Tranny.” 6. Someone calling you a sexist slur, like “Bitch,” “Slut,” “Cunt,” “Ho” or “Thot.”  7. Someone saying things like, “Hey Baby,” “Mmmm Sexy,” “Yo Shorty,” “Mami/Mamacita,” “Give me a smile,” or similar comments in a way that is disrespectful and/or unwanted and/or made you feel unsafe.  8. Someone whistling, honking, making kissy noises, “Pssst” sounds, or leering/staring aggressively at you. | No | Ever | Yes | Yes | No | Yes, relation to victim |
| Solymosi, 2018 | London, UK | Women | Cross-sectional survey (n =450); non-probability | Yes | Paper; List of behaviours or acts | One item was asked containing a list of 8 different behaviours. Participants responded (yes / no).   “Have you ever experienced any incidents of unwanted sexual behaviour while waiting for or travelling on public transport in London?”   1. No, I have not 2. Staring or leering that made you feel intimidated 3. Someone following you on to or off of public transport 4. Vulgar gestures 5. Someone taking photos of you (which feel like they are for sexual purposes) without your consent 6. Sexually suggestive comments/jokes that offended you 7. Intrusive/ lewd comments 8. Unwelcome touching (without your permission) in a sexual way, such as rubbing, kissing or groping 9. Flashing, indecent exposure, public masturbation | No | Ever | N/A | Yes | No | No |
| Whitfield, 2019 | Colorado, USA | Male and female LGBQ adults | Cross-sectional survey (n = 3690); non-probability | No | List of behaviours or acts | Participants were asked one item  “In the last 12 months, please indicate how often, if at all, you experienced homophobia, transphobia, and/or harassment.” The response alternatives allowed participants to select the location where the harassment occurred (one option was on the streets), the perpetrator, and the frequency. The response alternatives for frequency was: never; yearly; monthly; weekly; daily; more than once a day; not applicable. | No | Previous 12 months | Yes | Yes | Yes | Yes, occupation of the perpetrator |

*See Supplementary File 4 – Street Harassment Definitions
