# Supplementary material for: Operationalizing Street Harassment Using Survey Instruments: A Systematic Review of Measuring Harassment in Public Spaces Using Surveys
Source: Trauma Violence Abuse. 2024 Feb 5;25(4):2609–21. doi: 10.1177/15248380231219258 (PMC11370194; doi:10.1177/15248380231219258)
Supplement: sj-docx-5-tva-10.1177_15248380231219258 – Supplemental material for Operationalizing Street Harassment Using Survey Instruments: A Systematic Review of Measuring Harassment in Public Spaces Using Surveys [file sj-docx-5-tva-10.1177_15248380231219258.docx]

**Appendix E – Definitions of Street Harassment**

Definitions have been copied verbatim from articles, page numbers have been provided in the first column.

| **Reference** | ***Definition*** |
| --- | --- |
| Agrawal et al., 2020, p. 5 | *Sexual harassment, broadly defined, is “any unwanted attention including lewd comments, leering, sexual invitations, threats, displaying pornographic material, being followed or pictured, and public masturbation,” while sexual assault refers to situations “when someone is threatened, coerced, or forced into non-consensual sexual acts (Gekoski et al., 2017).” Scholars identify three categories of sexual harassment crimes in public spaces and transit environments: verbal, non-verbal, and physical (Ceccato et al., in press).* |
| Alam at al., 2010, p. 455 | *Gender harassment refers to a broad range of verbal and nonverbal behaviour aimed to insult or degrade rather than to win sexual favour. Unwanted sexual attention refers to a full range of verbal and non-verbal behaviour that is offensive, unwelcome, unreciprocated, and sexual. Sexual coercion refers to sex for favour and sexual intimidation.* |
| Betts et al., 2019, p. 38 | *Street harassment represents “unwanted behaviours from strangers that typically occur in public spaces (e.g. in the street or on public transportation)” (Davidson al., 2016, p. 553). According to Davidson et al., these behaviours include: whistling; leering; sexist, homophobic, or transphobic slurs; persistent requests for someone’s name or number after they have said no; sexual names; comments; and demands. Therefore, street harassment encompasses seemingly innocent comments to vulgar suggestions to outright threats which, along with the fear these behaviours generate, led Kissling (1991) to argue that street harassment contributes to a culture of “sexual terrorism” (p. 456). Moreover, Kearl (2010) proposed that street harassment represents a form of bullying behaviour that is motivated by power and disrespect with commonalities evident in street harassment and bullying behaviours. For example, name calling and comments are characteristic of verbal bullying (Rivers and Smith, 1994).* |
| Carretta et al., 2020, p. 526 | *Stranger harassment, also referred to as street harassment, includes women’s experiences of unwanted sexual attention from strangers in public settings such as sidewalks, parks, restaurants, and bars (Fairchild and Rudman2008). It encompasses behaviours ranging in severity from verbal stranger harassment (e.g., whistling, catcalling, sexist slurs, sexually explicit jokes, comments, or demands) to physical stranger harassment (e.g., unwanted touching, groping, pinching, hugging, rubbing or brushing against).* |
| Ceccato et al., 2021, p. 108 | *Following Breiding et al. (2014), we use the umbrella-term “sexual violence” in this study to cover a variety of sexual behaviours. These include (1) nonverbal sexual violence/abuse, such as stalking, exhibitionism, showing sexually explicit pictures, or making sexual gestures; (2) verbal sexual violence/abuse, such as sexual comments, jeering or taunting, and asking questions about sexual activity, and finally (3) physical sexual violence/abuse, which may involve behaviours such as touching, kissing, and rape.* |
| Ceccato et al., 2022, p. 27 | *Sexual harassment is a multifaceted phenomenon that encompasses a variety of sexual behaviours. In this study, these include nonverbal sexual violence/abuse, such as stalking, exhibitionism, showing sexually explicit pictures, or making sexual gestures; verbal sexual violence/abuse, such as sexual comments, jeering or taunting, and asking questions about sexual activity; and physical sexual violence/abuse, which may involve behaviours such as touching, kissing, and more serious offenses such as rape.* |
| Davidson et al., 2015, p. 54 | *According to Bowman (1993, p. 523), stranger harassment can be defined as ‘‘both verbal and nonverbal behavior, such as wolf-whistles, leers, winks, grabs, pinches, catcalls, and stranger remarks; the remarks are frequently sexual in nature and comment evaluatively on a woman’s physical appearance or on her presence in public.’’ That is, stranger harassment is perpetrated by individuals whom the victim does not know personally and occurs in public areas, including (but not limited to) on the street, on public transportation, and/or in bars and shops (Fairchild & Rudman, 2008).* |
| Davidson et al., 2016, p. 1 | *The term street harassment has been used to describe unwanted behaviours from strangers that typically occur in public spaces (e.g., the street, public transportation). More specifically, the organization Stop Street Harassment (2015) defines street harassment as including “unwanted whistling, leering, sexist, homophobic or transphobic slurs, persistent requests for someone’s name, number or destination after they’ve said no, sexual names, comments and demands, following, flashing, public masturbation, groping, sexual assault, and rape.”* |
| del Mar Rodas-Zuleta et al., 2022, p. 4 | *Street harassment: Unauthorized comments about another person’s appearance, in a public space, which do not necessarily contain sexual innuendo. • Sexual harassment: Using verbal, non-verbal, or written language, towards others for non-consensual sexual purposes only. • (Included for comparison) Sexual abuse or assault: A non-consensual sexual act, committed through violence.* |
| DelGreco et al., 2020, p. 474 | *Street harassment is generally defined as unwanted sexual attention, harassment, or objectification by a stranger in a public space, such as streets, parks, or public transportation (Bowman 1993; Wesselmann and Kelly 2010). Behaviours considered street harassment can include catcalling, stalking, gesturing, groping, exposing, or any other unwanted behaviours that attempt to sexualize the victim and make her feel unsafe or uncomfortable (Stop Street Harassment 2019).Although some street harassment behaviors can be vulgar, offensive, or explicitly threatening, many street harassment behaviours involve a man who is a stranger, addressing, greeting, expressing astonishment or admiration, summoning, and/or asking rhetorical questions with regard to a woman with whom he interacts in a public space (Bailey 2016).* |
| DelGreco et al., 2021, p. 1403 | *Street harassment is a component of sexual harassment, which is legally defined as unwelcome sexual advances, requests for sexual favours, or other verbal or physical conduct of a sexual nature (U. S. Equal Employment Opportunity Commission, 2019). Psychologically, it is more broadly defined as any form of unwanted sexual attention (Ilies et al., 2003; McMaster et al., 2002). By definition, all street harassment is not desired and it attempts to sexualize the receiver and make her feel uncomfortable. In other words, street harassment is unwanted sexual attention.* |
| Fairchild et al., 2008, p. 339 | *Stranger harassment is the ‘‘[sexual] harassment of women in public places by men who are strangers’’ (Bowman, 1993, p. 519). In other words, stranger harassment is perpetrated by men who are not known to the victim (i.e., not a co-worker, friend, family member, or acquaintance) in public domains such as on the street, in stores, at bars, or on public transportation.* |
| Fairchild, 2010, p. 192-193 | *Stranger harassment or street harassment can be defined as the “[sexual]harassment of women in public places by men who are strangers” (Bowman, 1993, p. 519) and includes “both verbal and nonverbal behaviour, such as wolf-whistles, leers, winks, grabs, pinches, catcalls, and street remarks; the remarks are frequently sexual in nature and comment evaluatively on a woman’s physical appearance or on her presence in public” (p. 523). While being the recipient of any of the above behaviours may indicate one has been stranger harassed, like sexual harassment, it is the perception of the target or victim that determines if the event was indeed harassing. Sexual harassment researchers have noted that the official definition of sexual harassment provided by the Equal Employment Opportunity Commission (EEOC) defines sexual harassment in terms of the perception of the victim in regard to frequency, coerciveness, and welcomeness (Faley, 1982; Pryor, 1985; Katz et al., 1996; Golden et al., 2001).* |
| Ferrer-Perez et al., 2021, p. 2 | - 1. *Conceptualisation of Street Sexual Harassment (SSH*   *A significant number of studies on this subject have focused on its conceptualization. Thus, in recent decades, numerous definitions have been proposed, with significant variations in the emphasis placed on the different elements that comprise SSH and their specifications [16]. As an example, Bowman [8] defined SSH as “sexual harassment against women in public spaces made by unknown men” (p. 51), and, more recently, SSH has been defined as “unwanted comments, gestures, or actions forced on a stranger in a public space without their consent, directed at them because of their actual or perceived sex, gender, gender expression, or sexual orientation” [17]. In general, all of these definitions include certain common dimensions that allow SSH to be characterized in the following way [2,3,6,8,10,16,18–21]:*   1. *Harassment occurs in a public or semi-public space (street, public transport) and is contextualized by a face-to-face interaction between two unknown people, that is, people who share no stable, long-term or safe connection.* 2. *Even though stalking, as Lopez [2] points out, is also a type of SSH, this type of violence tends to occur within a brief or even fleeting interaction (which not only constitutes one of its main characteristics, but also differentiates it from other forms of violence, such as sexual harassment in the workplace or academic environment).* 3. *The fleeting nature of this behaviour and the source of anonymity from which it occurs hinder its evaluation and criminal prosecution [22,23].* 4. *The absence of an intimate or other relationship causes the behaviour of the harasser to be perceived by the person harassed as an uncomfortable or even threatening transgression of her physical and psychological space.* 5. *The behaviour is unidirectional (meaning that neither the desires nor situation of the victim are taken into account) with a singular objective (meaning it is not meant to be public nor indiscriminate).* 6. *The assault primarily targets women, and the instigators are primarily men (alone or in a group), although SSH may also occur against men, especially those who do not conform to a heteronormative pattern [11,24], or against the non-binary population [25].* 7. *Although it may be considered a benign, harmless, or even normalized and socially tolerated act, it is an act of domination that impacts the sexual freedom and right to free movement of women, communicating the message that harassers have the right to occupy public spaces and to control, assault, or injure women. It therefore assumes the imposition of the desires of one (or a few) over another (or others) and has asexual connotation that is degrading and that objectifies, humiliates, and threatens the woman (or women), provoking in her (them) discomfort or fear.* 8. *It may also include visual assault (leering), nonverbal assault (sexual and obscene gestures, sighs, whistles or noises), verbal assault (jeering, sexual comments, whether supposedly positive, offensive or insulting), and/or assault in the sense of physical invasion of privacy (exhibitionism, public masturbation, groping.* |
| Fileborn, 2019, p. 223 | *Although a diffuse array of experiences fall under the banner of “street harassment,” definitions commonly include actions such as staring, ogling, wolf-whistling, unwanted verbal comments, following someone, car-horn honking, and groping, while some definitions also include sexual assault and rape (Gardner, 1995; Logan, 2015; Vera-Gray, 2016). What constitutes street harassment is highly context-dependent, with these practices perhaps best understood through their function, rather than form (Fileborn & Vera-Gray, 2017; Vera-Gray, 2016)* |
| Gurrola-Peña et al., 2022, p. 51 | *"a form of gender-based violence of a non-reciprocal sexual nature directed mainly against women. Although it is true that men are also exposed to this type of harassment, they are exposed to it to a lesser extent [5]. On the other hand, Arancibia [4] defined sexual street harassment as any practice that: a) has a sexual connotation, b) is perpetrated by strangers, c) is carried out in public or semi-public spaces, d) is unidirectional, and e) has the potential to produce discomfort (anger, fear, disgust, helplessness, stress, etc.)"* |
| Imtiaz et al., 2021, p. 1809 | *Despite the broad array of approaches that define sexual harassment, many researchers believe it is a psychological experience based on sexual unwanted touch, disrespectful comments, and threatening for sexual favours (Topa et al. 2008).* |
| Infante-Vargas et al., 2022, p. 218 | *Macmillan, Nierobisz, and Welsh (2000) categorize unwanted physical contact, verbal comments, ogling and stalking as stranger harassment that is typical in public places such as streets. This type of stranger harassment can also be encountered on public transportation (Fairchild & Rudman, 2008) and it may include actions such as catcalling, wolf-whistling and groping. These forms of gender violence can be the beginning of an interaction that results in even more serious harm, such as rape or murder* |
| Jabeen et al., 2017, p. 91 | *Street harassment may represent the most common and frequent type of sexual harassment encountered by women whereas sexual harassment includes unwanted sexual actions in the public places, particularly on roads, on public transport and at work places. (Joseph, et al., 2006)* |
| Kash, 2019, p. 235 | *Sexual assault and harassment encompass an array of behaviours that mainly target women (Stringer, 2007; Clark et al., 2016; Madan and Nalla, 2016). These acts can be verbal (sexual comments or intrusive questioning), physical (touching, fondling, groping, rape), or based on exposure (exhibitionism, showing pornography). Here, I define sexual assault as any form of unwanted physical contact, and harassment as non-contact forms of sexual aggression.* |
| Kearl, 2014, p. 5, 8-9 | *“Street harassment” describes unwanted interactions in public spaces between strangers that are motivated by a person’s actual or perceived gender, sexual orientation, or gender expression and make the harassee feel annoyed, angry, humiliated, or scared. Street harassment can take place on the streets, in stores, on public transportation, in parks, and at beaches. It ranges from verbal harassment to flashing, following, groping, and rape. It differs from issues like sexual harassment in school and the workplace or dating or domestic violence because it happens between strangers in a public place, which at present means there is less legal recourse. (p. 5)*  *Examples of street harassment ‘include: honking and whistling, calling out phrases like ‘hey baby, ‘hey shorty’, and ‘mamacita’, often referred to as ‘catcalling’, persistent requests for a name, numbers, or date after being denied, ignored, or otherwise informed that the recipient isn’t interested, sexist comments and telling someone to smile, evaluative comments both ‘positive’ like ‘nice legs’ and ‘negative’ like ‘fat cow’, sexually explicit comments or demands, homophobic or transphobic slurs, vulgar gestures and ‘pssst’ sounds, following, flashing or public masturbation, grabbing and rubbing against someone, sexual assault’. Street harassment happens without the consent of the harassee and is done with a sense of entitlement or disrespect for that person, as if the harasser has the right to comment on, touch, or follow the harassed person. (p. 8-9).* |
| Khairat, 2016, pp. 91-92 | *Harassment is defined as "a negative behaviour that annoys visitors". In addition, it is clarified from the viewpoint of the tourist as "any annoying behaviour taken to the extreme". However, from the viewpoint of the host perpetrator, "harassment is simply refusing to get "no" for an answer".   Furthermore, harassment is "words, or actions (usually repeated or persistent) that, annoy, alarm, or causes substantial emotional distress in that person and serves no legitimate purpose".   Harassment is "a form of discrimination and a gross violation of a person’s human rights and human dignity that is unwelcome"  Also, harassment is defined as "any unwanted conduct of a sexual nature having the effect of verbal, non-verbal, visual, psychological, or physical harassment". Moreover, harassment is referred to as the "highly symbolic form of violence which is experienced by women in the form of heckling, whistling, rating, propositioning, leering, fondling, and in other ways assaulting and humiliating by men as they go about their daily lives in public spaces"  Harassment can be classified into a number of main categories: verbal harassment, non-verbal/gestural harassment, physical harassment, pestering, begging, drug peddling, and sexual harassment argue these incidents range from petty harassment by vendors to robberies and on to more serious and violent crime and even murder* |
| Lebugle, 2017, p. 1, note 1 | *In law, sexual harassment is defined as repeatedly subjecting a person to language and behaviour of a sexual nature which either compromise his or her dignity through their degrading or humiliating nature, or which create a situation that is intimidating, hostile or offensive to the victim. The use of any form of severe pressure, even if not repeated, with the real or apparent aim of obtaining a sexual act, be it for the perpetrator or for a third person, is also considered as sexual harassment* |
| Loukaitou-Sideris et al., (2022), p. 177 | *“improper behaviour that has a sexual dimension” (O’Donohue et al. 1998, p. 112), but an array of different behaviours falls within this broad definition. Elsewhere, we have classified these behaviours into three broad categories: verbal harassment, physical harassment (involving touching), and non-verbal/non-physical harassment (e.g., sexual looks or gestures, stalking, indecent exposure) (Ceccato and Loukaitou-Sideris 2020)* |
| Loukaitou-Sideris et al., 2022, p. 2 | *We use the term “sexual harassment” to indicate “any unwanted attention including lewd comments, leering, sexual invitations, threats, displaying pornographic material, being followed or pictured, and public masturbation” (Gekoski etal. 2017, 4). Ceccato and Loukaitou-Sideris (2020) identify three categories of sexual harassment in transit environments: verbal, physical, and nonverbal/nonphysical* |
| Macmillan et al., 2000, p. 307 | *"...a range of behaviours including sexual comments, unsolicited and unwanted touching, and attempts to coerce an individual into complying with sexual demands." and "...behaviours such as unwanted physical contact, verbal comments, ogling, and stalking, stranger harassment is typical of public places such as streets."* |
| Mellgren et al., 2018, p. 263 | *According to the World Health Organization (WHO; 2013) violence against women is defined as “any act of gender-based violence that results in, or is likely to result in, physical, sexual or mental harm or suffering to women, including threats of such acts, coercion or arbitrary deprivation of liberty, whether occurring in public or in private life” (p. 2). Further, according to WHO (2013), “sexual violence is any sexual act, attempt to obtain a sexual act, or other act directed against a person’s sexuality using coercion, by any person regardless of their relationship to the victim, in any setting” (p. 2). Sexual violence, according to this definition, can include acts ranging from name-calling and minor forms of sexual harassment to violent offenses such as rape and homicide.* |
| Mishra et al. 2018, pp. 20-21 | *Sexual harassment against women is an incidence of violation of personal space of women by individual or groups of men where they do uninvited sexual advances, unwelcomed gestures, comments, insults, slurs and other obscenities at women in public places. It can exist as any form of sexual contact or activity ranging from verbal abuse to physical assault.2The following are the behaviours which could be considered as sexual harassment when they are unwelcomed: Verbal: Whistling or making cat calls at someone; making sexual comments about a person’s clothing or body; telling sexual jokes or stories Non-verbal: Paying unwanted attention to someone (i.e., staring, following, blocking a person’s path) displaying sexually suggestive visuals; making facial expressions such as winking, throwing kisses, or licking Physical: Hanging around, standing close, or brushing up against a person; touching a person’s clothing, hair, or body touching or rubbing oneself sexually around another person; hugging, kissing, patting or stroking* |
| Mora et al., 2022, p. 19 | *Gender‐based harassment consists of unsolicited verbal and nonverbal sexual advances, requests for sexual favours, or physical behaviours of a sexual nature (Eom et al., 2015). Additionally, gender‐based harassment includes unwelcome physical contact as well as sexual jokes, comments, or gestures (Young et al.,2009).* |
| Moreno et al., 2022, p. 159 | *Among the behaviours that could be classified as “street harassment” are: whistling, staring, making sexually explicit comments, grunting, making offesive comments about the body, honking the horn of a car, exhibitionism, or masturbation on public transport or in the streets (De Luna-Meza, 2013; Fileborn & Vera-Gray, 2017). These behaviours share the common characteristic that the aggressor is unknown to the victim, which makes the experience particularly difficult to anticipate and, therefore, avoid (Gaytán, 2007; MacMillan et al., 2000; Observatorio contra el Acoso Callejero de Chile, 2015)* |
| Reed et al., 2019, p. 1 | *Sexual harassment, defined as unwanted and unwelcome behaviour of a sexual nature, is a form of sexual violence that is extremely prevalent among youth (Chiodo, Wolfe, Crooks, Hughes, & Jaffe, 2009; Clear et al., 2014; Hill & Kearl, 2011).* |
| Saunders et al., 2017, p. 325 | *Examined originally under the umbrella of sexual harassment (Fitzgerald1993; Rotundo et al.2001), stranger harassment resembles the kind of sexual harassment that women experience in the workplace in that it involves unwanted verbal (e.g., catcalls, whistling, hollering)or non-verbal (e.g., following, leering, grabbing) sexual attention (Bowman1993;Davidsonetal.2015; Fairchild andRudman2008;Gardner1995; Wesselmann and Kelly2010).But stranger harassment differs from traditional sexual harassment in some important ways. First, stranger harassment occurs, by definition, between two people who are unknown to each other in public (Fairchild and Rudman2008; Fairchild2010). Accordingly, women might encounter stranger harassment in any number of public settings, including activities like walking down the street, waiting for or traveling via public transportation, shopping in stores, or patronizing restaurants or bars (Fairchild and Rudman2008). Second, unlike sexual harassment—which often occurs in professional or academic contexts with an implied quid pro quo element (Fairchild andRudman2008; Fitzgerald1993; Gregusetal.2014;McCartyet al.2014)—stranger harassment often occurs without a clear purpose or agenda, as the contact between the two parties tends to last only moments and is often one-sided.* |
| Shibata, 2020, p. 293 | *This groping involves touching or pressing one’s body against another person inappropriately in a public place or on a public vehicle.* |
| Smith, 1994, pp. 117-118 | *"As for victim-offender relationships, I classified an incident as stranger violence if the women did not see or recognise the offender, knew him only by sight, or knew him only by hearsay"* |
| Smith et al., 2022, p. 1965 | *Sexual harassment includes unwanted sexual attention or contact, as well as harassment based on sex/gender or sexual orientation (i.e. gender harassment), with experiences ranging in physical severity and inclusive of forced sexual interaction (Fitzgerald et al., 1995)* |
| Solymosi et al., 2018, p. 572 | *The World Health Organisation provides a broad definition of sexual violence as “Any sexual act, attempt to obtain a sexual act, unwanted sexual comments or advances, or acts to traffic, or otherwise directed, against a person’s sexuality using coercion, by any person regardless of their relationship to the victim, in any setting, including but not limited to home and work” (MOPAC & NHS England 2016: pp. 19–20). It can take place anywhere, including the workplace, schools, streets, public transport and social situations. It includes flashing, obscene and threatening calls, and online harassment. In particular here, we focus on sexual harassment as the unwanted verbal or physical conduct of a sexual nature, which occur in transport setting* |
